# The p53 tumor suppressor modulates the expression of proteins that control natural killer cell activity

**DOI:** 10.1186/s12964-026-02772-9

**Published:** 2026-03-05

**Authors:** Agnieszka Będzińska, Agnieszka Gdowicz-Kłosok, Małgorzata Krześniak, Barbara Łasut-Szyszka, Marcin Zeman, Agata Chwieduk, Andrzej Smagur, Jadwiga Jablonska, Marek Rusin

**Affiliations:** 1 Center for Translational Research and Molecular Biology of Cancer, Maria Skłodowska-Curie National Research Institute of Oncology, Gliwice Branch, ul. Wybrzeże Armii Krajowej 15, Gliwice, 44-101 Poland; 2 III Department of Oncological Surgery, Maria Skłodowska-Curie National Research Institute of Oncology, Gliwice Branch, Gliwice, 44-101 Poland; 3 Department of Bone Marrow Transplantation and Hematology-Oncology, Maria Skłodowska-Curie National Research Institute of Oncology, Gliwice Branch, Gliwice, 44-101 Poland; 4 Department of Otorhinolaryngology, University Hospital Essen, University Duisburg-Essen, Essen, 45147 Germany; 5 German Cancer Consortium (DKTK) Partner Site Düsseldorf/Essen, Essen, 45147 Germany

**Keywords:** Innate immunity, SLAMF7, p53, Natural killer cells, Paclitaxel

## Abstract

**Background:**

Natural killer (NK) cells are at the forefront of the fight against infections and cancer, where they recognize targets through a system of inhibitory and activating ligands expressed on monitored cells. The transcriptional regulation of such ligands in tumors remains poorly understood. Our previous transcriptomic studies suggest that the tumor suppressor p53 may upregulate key NK-activating ligands, including SLAM family member 7 (*SLAMF7*) and natural killer cell cytotoxicity receptor 3 ligand 1 (*NCR3LG1*). Herein, we aimed to validate the functional role of this regulatory axis.

**Methods:**

Isogenic p53-proficient and p53-deficient cell lines from diverse cancer types (A549, NCI-H460, U-2 OS) were used to examine p53-dependent regulation of *SLAMF7* and *NCR3LG1.* Luciferase reporter assays assessed putative p53-responsive enhancer elements, with pharmacological activation of p53 achieved using DNA-damaging agents (actinomycin D and camptothecin) and the MDM2 proto-oncogene inhibitor nutlin-3a. The functional impact of NK cell-mediated cytotoxicity was assessed using primary human NK cells and the NK-92 cell line.

**Results:**

Pharmacological activation of p53 using camptothecin or combined actinomycin D and nutlin-3a strongly induced *SLAMF7* and *NCR3LG1* expression in p53-proficient, but not p53-deficient, cancer cell lines. The SLAMF7 protein, typically expressed only in immune cells, was strongly induced in epithelial and mesenchymal cancer models following p53 activation. Paclitaxel upregulated SLAMF7 independently of p53, suggesting an alternative pathway of induction. Notably, dual treatment with actinomycin D and nutlin-3a induced the secretion of soluble SLAMF7. Also, *NCR3LG1* was strongly upregulated in a p53-dependent fashion. The cloned fragments of these genes, located in regions suggestive of enhancer activity, conferred p53 responsiveness to the reporter gene. Functionally, dual p53 activation by actinomycin D and nutlin-3a significantly increased tumor cell susceptibility to cytolysis by both primary human NK cells and the NK-92 cell line.

**Conclusions:**

SLAMF7 expression can be induced in non-hematological cancers by p53-dependent and p53-independent mechanisms following exposure to anticancer agents. Given that SLAMF7 is a clinically validated target of the monoclonal antibody Elotuzumab in multiple myeloma, its drug-inducible expression in solid tumors is of particular interest. This finding may support the development of novel therapeutic strategies incorporating SLAMF7-targeting antibodies in p53-competent non-hematologic malignancies.

**Supplementary Information:**

The online version contains supplementary material available at 10.1186/s12964-026-02772-9.

## Introduction

The p53 tumor suppressor protein activates several forms of regulated cell death, including apoptosis, necroptosis, ferroptosis, and autophagy [1], each of which is executed by proteins within the dying cells. However, cell death may also be triggered by the close interaction between the target cells destined for destruction and the effector cells that induce it. Such cells include immune cells, particularly cytotoxic T lymphocytes (CTLs) and natural killer (NK) cells, which target cancerous or infected cells. Notably, p53 has also been shown to promote this form of cell death (elimination). For instance, p53 promotes the expression of the FAS receptor, which initiates apoptosis when activated by its ligand expressed on the surface of cytotoxic lymphocytes, including NK cells. The intrinsic resistance of many cancer cells to apoptosis induced by the FAS ligand can be overcome by strong activation of p53 [2].

NK cells are immune cells derived from the lymphoid lineage and are active in the early phase of infection or other stress conditions. Two sets of signals, positive and negative, govern the interaction between NK cells and their potential targets, with negative signals reducing and positive signals increasing the likelihood of cell death. The outcome of the encounter between a target cell and an NK cell is determined by the balance of the signals on the surface of the former [3].

The upregulation of ligands that activate NK receptors represents a logical strategy to mark cancer cells for immune-mediated destruction. Interestingly, two research groups found that p53 can induce the expression of the gene encoding UL16 binding protein 2 (ULBP2), a ligand that activates the natural killer group 2, member D (NKG2D) receptor [4, 5]. These findings suggest that cellular stress leading to p53 activation may enhance NK cell elimination of transformed cells.

Transcriptomic analysis of cancer cells exposed to actinomycin D (ActD) and nutlin-3a (Nut3a) (A + N) - compounds that synergistically activate p53 - revealed that p53 may upregulate several genes involved in promoting NK cell cytotoxicity, including SLAM family member 7 (*SLAMF7*) [6, 7]. Unlike typical receptor-ligand interactions, SLAMF7 is expressed on both NK cells and target cells, where it mediates homophilic binding or self-ligation [8]. When SLAMF7 on NK cells binds to SLAMF7 on cancer cells (e.g., multiple myeloma [MM] cells), an activation signal is sent that promotes the killing of the cancer cell. Hence, one may expect that any stress factor that promotes the expression of SLAMF7 molecules on cancer cells makes them a conspicuous target for NK cells. Indeed, this mechanism has been experimentally demonstrated in HeLa cells that expressed SLAMF7 ectopically. HeLa cells growing in control conditions do not express SLAMF7 [9]. As such, this strategy appears to be effective only in hematological cancer cells, as SLAMF7 was expressed only on leukocytes.

Another innate immunity-related gene strongly upregulated by A + N is natural killer cell cytotoxicity receptor 3 ligand 1 (*NCR3LG1*), which encodes the ligand for the NKp30 activating receptor [10]. Typically low in healthy tissues, *NCR3LG1* is upregulated in various tumors [11]. Here, we aimed to assess if these two genes are directly regulated by p53 and what impact it has on the destruction of cancer cells by NK cells.

## Results

### SLAM family member 7 protein is upregulated in cancer cell lines in response to various p53-activating agents

Our transcriptomic analysis of A549 cells demonstrated that treatment with A + N significantly upregulates the expression of *SLAMF7* [6]. A more detailed study demonstrated that A + N cooperate to upregulate this gene, and that their combined action also induces *SLAMF7* expression in A375 (melanoma cell line) and U-2 OS (osteosarcoma cell line) [7].

**Fig. 1:**
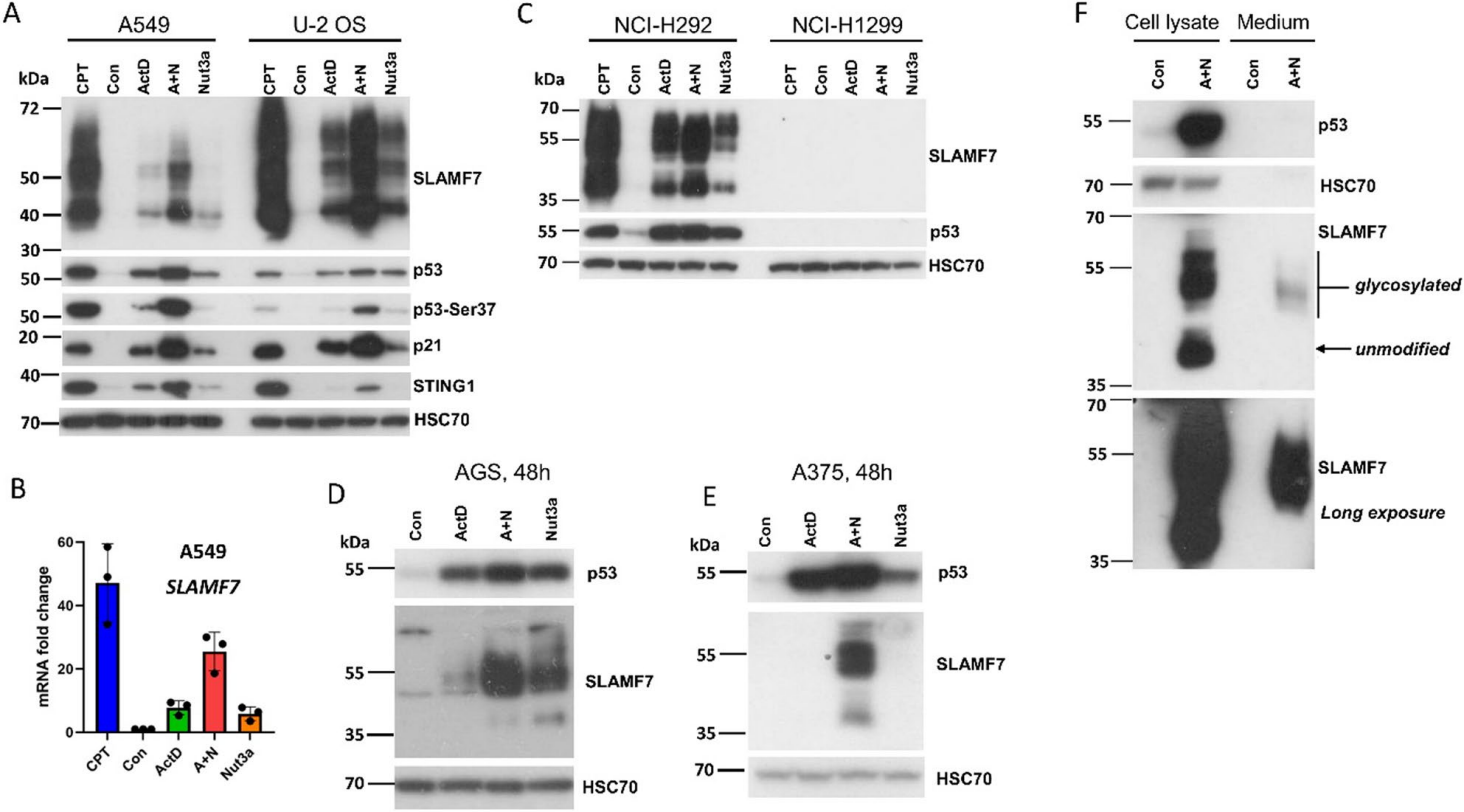
Various p53 activators induce SLAM family member 7 expression in cancer cell lines. **A**, **C**,** D**, and **E**. Protein levels of p53, SLAM family member 7 (SLAMF7), p53-Ser37, p21, STING1 and the loading control (HSC70) in the indicated cell lines after 48 hours of treatment with actinomycin D (ActD), nutlin-3a (Nut3a), their combination (A+N), camptothecin (CPT), or mock-treated (Con). **B**. Fold change in SLAMF7 mRNA levels in A549 cells treated as shown for 30 hours. The graph was prepared based on our published RNA-Seq data [7]. **F**. SLAMF7 expression was analyzed in cell lysate and concentrated culture medium from either Con cells or those exposed to A+N for 48 hours. Protein levels were assessed by Western blotting. The two lower panels represent the same blot with short (upper) and long (lower) exposure times to enhance visualization of the bands

This work began by testing whether the upregulation of *SLAMF7* at the mRNA level leads to the accumulation of its protein. We exposed A549 and U-2 OS cells to camptothecin (CPT), ActD, Nut3a, and A + N, as demonstrated in Fig. 1A. The upregulation of the SLAMF7 protein was more pronounced in cells exposed to the anticancer drug CPT than in those treated with A + N. A similar trend was observed for the induction of *SLAMF7* mRNA, as shown by our previously published transcriptomic data (Fig. 1B). ActD and Nut3a, when used alone, were modest inducers; however, they cooperated to upregulate *SLAMF7* expression in both cell lines. The Western blot presented on Fig. 1A also shows the expression of p53 with phosphorylated serine 37, which serves as p53 activation marker. Moreover, the expression of p21, which is coded by archetypal p53 target gene (*CDKN1A*) is also shown. The STING1 protein is encoded by another p53 target gene, which like SLAMF7, is involved in immunity [6, 7].

Several forms of SLAMF7 were visible on the Western blots. The calculated molecular size of the SLAMF7 protein is approximately 40 kDa (native form); therefore, the lower band, located near the 40 kDa marker, likely represents the unmodified SLAMF7 protein, while the bands above 40 kDa correspond to its post-translationally modified forms. These sizes are consistent with the size of glycosylated SLAMF7 [12]. Since the upper bands appear as a smear, it can be inferred that the SLAMF7 protein undergoes various patterns of glycosylation.

To further characterize SLAMF7 expression in cancer cell lines exposed to p53 activators, we employed an NCI-H292 lung cancer cell line expressing wild-type p53 and the p53-null NCI-H1299 lung cancer cell line. SLAMF7 expression in NCI-H292 cells was induced not only by A + N, but also by ActD or Nut3a when used individually (Fig. 1C). Neither compound induced SLAMF7 expression in NCI-H1299 cells, suggesting that *SLAMF7* is a p53-regulated gene. Additionally, SLAMF7 is upregulated in other cancer cell lines derived from gastric adenocarcinoma (AGS; Fig. 1D) and melanoma (A375, Fig. 1E). In the melanoma cell line, we observed a strong synergistic effect between ActD and Nut3a in the upregulation of SLAMF7. Thus, the SLAMF7 protein can be markedly upregulated in various cancer cell lines by A + N, as well as by CPT, and by ActD and Nut3a individually in some cell lines (NCI-H292, U-2 OS, AGS).

### The larger form of SLAM family member 7 is detected in the medium of cells exposed to actinomycin D combined with nutlin-3a

SLAMF7 is a single-pass transmembrane protein with an extracellular amino-terminal immunoglobulin-like domain [13]. The extracellular domain is cleaved by unidentified enzymes and detected as soluble SLAMF7 (sSLAMF7) in the serum of patients with MM, who express high levels of SLAMF7 [14, 15]. We hypothesized that the overexpression of SLAMF7 in cells exposed to A + N may be associated with the presence of sSLAMF7 in the culture medium of these cells. To test this, we examined the presence of SLAMF7 in cell lysates prepared from A549 cells exposed to A + N and in the concentrated medium from the same cells (Fig. 1F). Three major forms of SLAMF7 were detected In the lysates of treated cells, including the unmodified 40 kDa form and at least two larger versions (likely glycosylated) at approximately 50 and 57 kDa. However, only the larger forms were detected in the concentrated medium. Images were taken at two exposure times; even on the overexposed film, only the larger forms were visible. This suggests that the SLAMF7 detected in the medium is shed rather than leaked from the dying cells – if leakage were occurring, both unmodified and modified forms of SLAMF7 would be expected to be present in the medium.

### The p53 protein participates in upregulation of SLAM family member 7

**Fig. 2:**
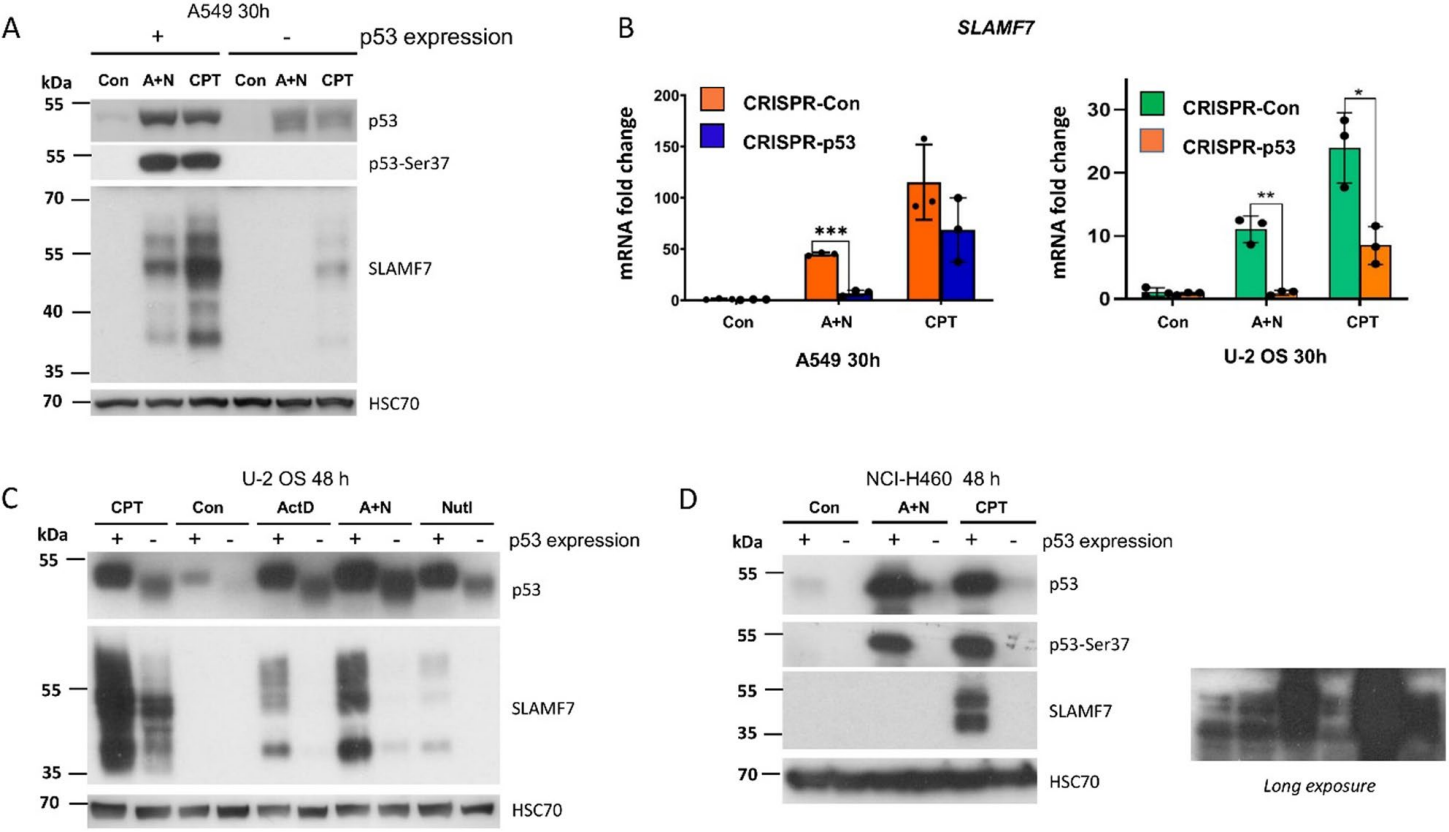
SLAM family member 7 expression is reduced in p53-deficient cells. **A**. Western blot analysis of indicated proteins in p53-proficient and p53-deficient A549 cells treated with the specified compounds for 30 hours. **B**. Relative SLAM family member 7 (SLAMF7) mRNA levels measured by semi-quantitative real-time polymerase chain reaction in p53-proficient and p53-deficient A549 and U-2 OS cells under the same treatment conditions. Statistical significance was calculated using a Student’s t-test (* *p* < 0.05, ** *p* < 0.01, *** *p* < 0.001). **C**. Protein expression in p53-proficient (+) and p53-deficient (-) U-2 OS. **D**. Protein expression in p53-proficient (+) and p53-deficient (-) NCI-H460 cells. The SLAMF7 panel is shown with both short and long exposure times to visualize the protein bands in actinomycin D (ActD) and nutlin-3a (Nut3a) (A+N)-treated cells better

The lack of SLAMF7 upregulation in the p53-null NCI-H1299 cell line suggests that p53 participates, directly or indirectly, in the activation of this gene. To further test this hypothesis, we employed cell lines with p53 mutations generated using CRISPR/Cas9 technology. The generation of these p53-deficient cells was described in our earlier report [6]. The plasmids used in the procedure target the p53 gene fragment, which codes for the amino-terminal region of the protein. The resulting double-strand break is repaired, sometimes generating in-frame deletions that produce a p53 molecule several kilobases shorter than the wild-type form. Using this approach, we generated a mixture of A549 and U-2 OS clones with inactivated p53 (Fig. 2A, C). Most clones do not express wild-type p53, as evidenced by the lack of signal with an antibody against phosphorylated Ser37, which is a marker of activated p53 (Fig. 2A). The *SLAMF7* gene expression was strongly attenuated in these p53-deficient A549 and U-2 OS cell lines, both at the mRNA (Fig. 2B) and protein level (Fig. 2A, C).

In the case of NCI-H460 cells, we also generated a mixture of clones that do not typically express p53. In this cell line, expression of SLAMF7 following treatment with either CPT or A + N was similarly lost (Fig. 2D). Together, these results obtained from three different p53-inactivated cell lines clearly demonstrate that this tumor suppressor plays a critical role in the upregulation of *SLAMF7* following treatment with p53-activating agents.

### Paclitaxel activates SLAM family member 7 independently of p53

**Fig. 3:**
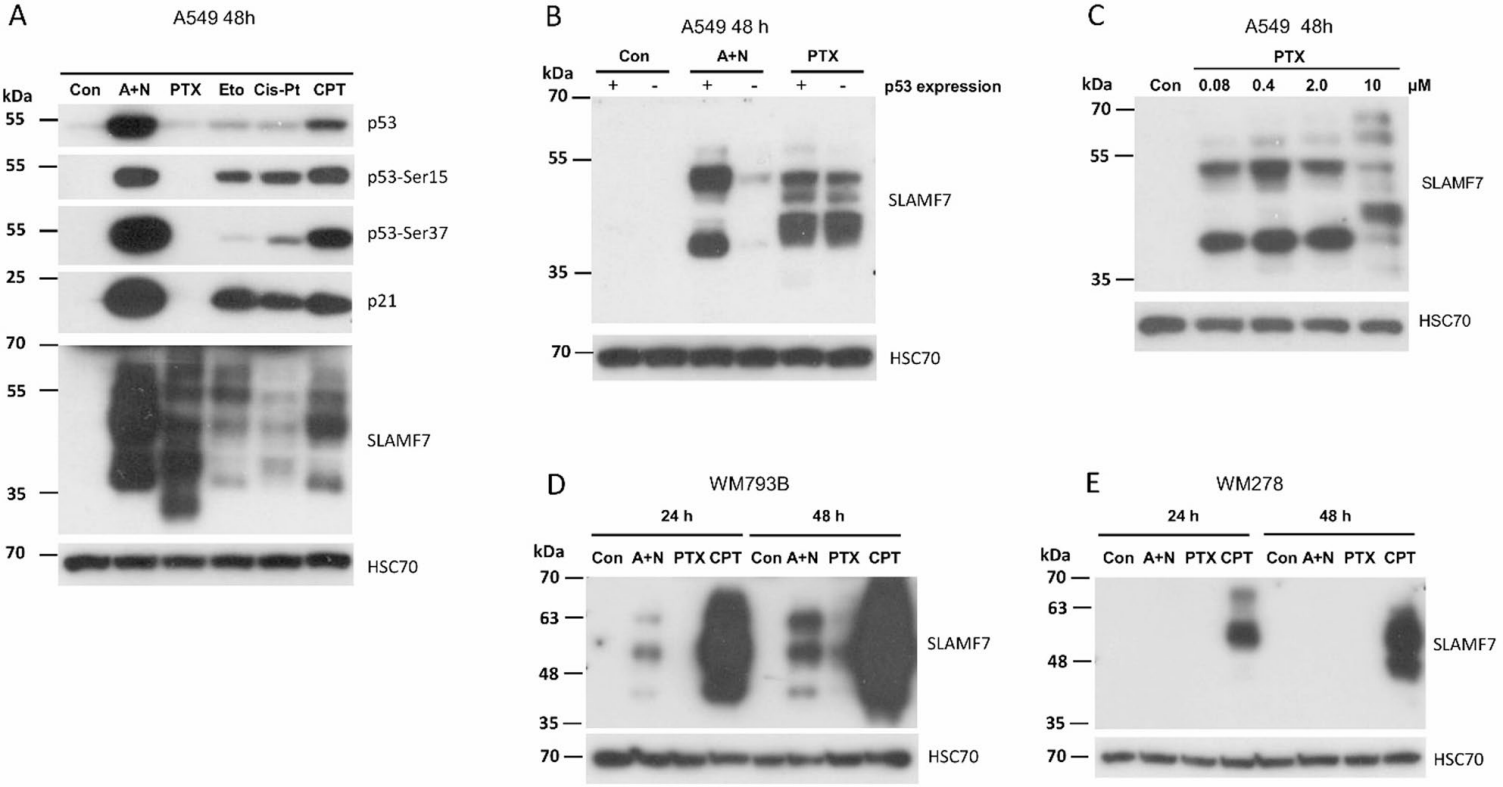
Paclitaxel induces SLAM family member 7 expression in a p53-independent manner.** A**. Expression of total and phosphorylated forms of p53 (p53-Ser15 and p53-Ser37) in A549 cells treated with actinomycin D and nutlin-3a (A+N), paclitaxel (PTX; 10 μM), etoposide (Eto; 15 μM), cisplatin (Cis-Pt; 10 μM), or camptothecin (CPT; 5 μM). **B**. SLAM family member 7 (SLAMF7) expression in p53-proficient (+) and p53-deficient (-) A549 cells following treatment with the indicated compounds. **C**. Dose-response analysis of SLAMF7 expression in A549 cells treated with increasing concentrations of PTX. **D**. **E**. SLAMF7 expression in two melanoma cell lines exposed to either A+N, PTX, or CPT

We demonstrated that SLAMF7 can be activated by the experimental drug combination (A + N), by CPT – a precursor of the established anticancer drugs topotecan and irinotecan - or by ActD and Nut3a acting individually in some cell lines. Next, we tested whether other anticancer agents can activate the expression of *SLAMF7* (Fig. 3A). We evaluated the effect of paclitaxel (PTX), etoposide (Eto), and cisplatin (Cis-Pt) in addition to A + N and CPT. All tested compounds were able to upregulate SLAMF7 expression.

To assess the degree of p53 activation, we examined not only total p53 level but also its phosphorylation at Ser15 and Ser37, as well as the expression of p21, a product of the p53 target gene cyclin-dependent kinase inhibitor 1 A (*CDKN1A*). The primary conclusion from this experiment is that all tested anticancer agents upregulated SLAMF7 expression. Surprisingly, in cells exposed to PTX, the upregulation of SLAMF7 was not accompanied by any signs of p53 pathway activation - no accumulation of total p53, no phospho-p53 detected, and no upregulation of p21. Interestingly, excluding PTX, SLAMF7 expression correlated better with phospho-Ser37 than with phospho-Ser15 of p53. This strongly suggests that PTX induces *SLAMF7* expression via a p53-independent mechanism, which has not been previously described.

To test this hypothesis directly, we treated p53-proficient and p53-deficient A549 cells with A + N and PTX and measured SLAMF7 expression (Fig. 3B). In p53-deficient cells, SLAMF7 expression following A + N treatment was very low compared to that in p53-proficient cells. However, with PTX, SLAMF7 expression was similar in both p53-proficient and p53-deficient cells. Interestingly, the smaller form in the PTX-treated cell migrated slightly higher than the corresponding form in A + N-treated cells.

In a dose-response experiment, we observed that even a very low concentration of PTX (80 nM) was sufficient to upregulate SLAMF7 (Fig. 3C), suggesting that PTX has the potential to upregulate SLAMF7 expression in cancer cells in vivo regardless of p53 status. Subsequently, we also tested melanoma-derived cancer cell lines for their ability to upregulate SLAMF7 in response to A + N, CPT, or PTX (Fig. 3D, E). Both melanoma cell lines have wild-type *TP53* [16]. We detected SLAMF7 upregulation in response to CPT in both cell lines, observed upregulation by A + N in one cell line, and no upregulation by PTX in either cell line. Thus, CPT induced SLAMF7 upregulation in all tested cell lines with functional p53 protein, whereas PTX-mediated SLAMF7 upregulation was p53-independent and cell type-specific.

### A putative p53-response element upstream of the SLAM family member 7 gene

**Fig. 4:**
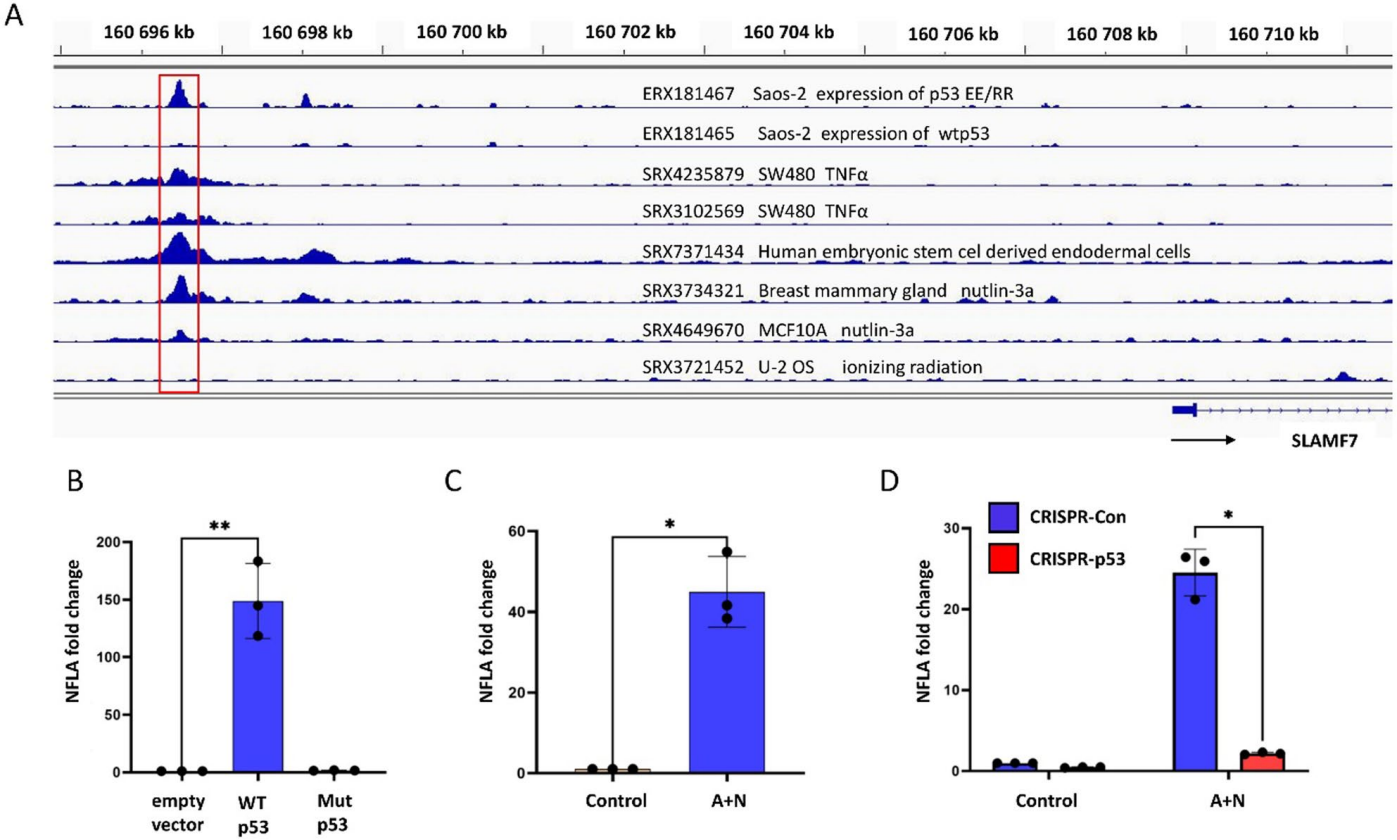
A DNA sequence located 12 kb upstream of the SLAM family member 7 gene functions as a p53-responsive element. **A**. Integrated Genome Viewer (IGV) snapshot of a p53 binding peak approximately 12 kb upstream of the SLAM family member 7 (SLAMF7) gene. Publicly available ChIP-Seq datasets from eight experiments in the indicated cell lines were retrieved via the ChIP-Atlas platform [17]; the red square marks the DNA fragment cloned into the pGL3-Basic reporter vector. The black arrow indicates the direction of SLAMF7 transcription. The IDs from the ChIP-Seq data are shown alongside short description samples. **B**. Relatively normalized firefly luciferase activity (NFLA) in U-2 OS cells co-transfected with reporter constructs containing the putative p53-responsive element and either an empty vector, a vector expressing wild-type p53, or a vector expressing the mutant p53 (Val143Ala). **C**. NFLA values in U-2 OS cells transfected with the reporter vector and cultured under control conditions or treated with A+N to activate the endogenous p53. **D**. NFLA values in p53-deficient (CRISPR-p53) and control (CRISPR-Con) U-2 OS cells transfected with the reporter vector and exposed to actinomycin D and nutlin-3a (A+N) or mock-treated. Data in **B**, **C**, and D represent means ± standard deviation from three independent biological replicates. Statistical significance was assessed using Student’s t-test (* *p *< 0.05, ** *p* < 0.01)

To test whether *SLAMF7* is directly regulated by p53, we searched the published ChIP-Seq data using the ChIP-Atlas tool [17] for the potential p53 binding sites within or near the *SLAMF7* gene. The closest site identified is located 12 kb upstream of *SLAMF7* (Fig. 4A). Although not commonly detected across cell types or treatment conditions, it is observed in mammary gland cells exposed to Nut3a, in SW480 cells exposed to tumor necrosis factor alpha (TNFα), and in p53-null Saos-2 cells transfected with a p53 mutant that enhances cooperative binding between p53 monomers [18]. These findings suggest that p53 can bind this site under specific conditions, and because this site lies far from the *SLAMF7* promoter, it likely functions as an enhancer.

It is well-established that the p53 protein can regulate its target genes by binding to distal enhancers [19]. To test the functionality of this candidate site, we cloned DNA fragments upstream of a luciferase reporter gene and co-transfected it to U-2 OS cells along with either an empty vector, a vector expressing wild-type p53, or a vector encoding a transcriptionally inactive p53 mutant (p.Val143Ala)(Fig. 4B). Notably, only the wild-type p53 robustly activated the reporter gene, resulting in an approximately 150-fold induction. These results support the hypothesis that this sequence is a p53-responsive enhancer regulating *SLAMF7* expression.

One caveat is that the enhancer was cloned without a promoter. By definition, the enhancers require promoters to initiate gene transcription; however, some enhancers can start transcription on their own, producing bidirectional enhancer RNAs (eRNAs), as shown by RNA polymerase II binding studies [20]. Interestingly, p53-bound enhancers can produce eRNAs in a p53-dependent manner [21], suggesting that p53-responsive enhancers may directly drive a reporter gene transcription.

In the next step, we assessed whether the endogenously activated p53 can stimulate the activity of the putative *SLAMF7* enhancer. U-2 OS cells were transfected with the *SLAMF7* reporter construct (coding for firefly luciferase) and a *Renilla* luciferase plasmid to control for the treatment-related stress. Subsequently, 24 hours’ post-transfection, cells were either maintained in a standard medium or treated with A + N. After another 24 h, firefly and *Renilla* luciferase activities were measured - presented on the graph as normalized firefly luciferase activity (NFLA, Fig. 4C). A + N treatment stimulated the activity of the reporter gene by more than 40-fold through the activation of endogenous p53. To confirm p53-dependence, we repeated the assay using p53-proficient and p53-deficient U-2 OS cells. Reporter activation was significantly lower in p53-deficient cells (Fig. 4D), further supporting p53’s role in enhancer activation.

**Fig. 5:**
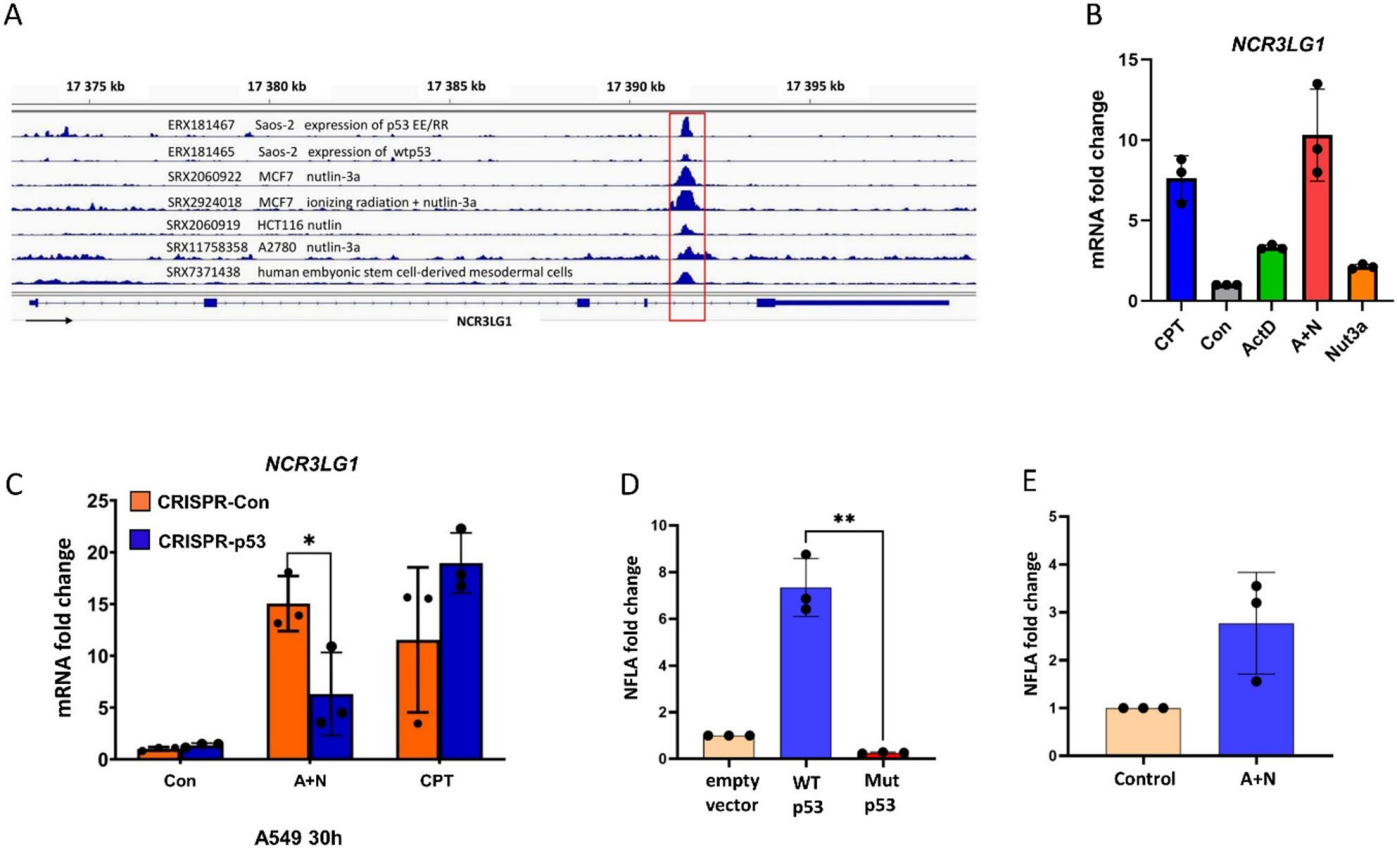
A DNA sequence located in intron 4 of the natural killer cell cytotoxicity receptor 3 ligand 1 gene acts as a p53-responsive element. **A**. Genome Browser (IGV) views showing p53 binding peaks within intron 4 of the natural killer cell cytotoxicity receptor 3 ligand 1 (NCR3LG1) gene. ChIP-seq coverage tracks from seven publicly available datasets (experiment accession number shown) were imported using the CHIP-Atlas tool [17]. The red square marks the DNA fragment cloned into the pGL3-Basic reporter vector encoding the firefly luciferase gene. The black arrow indicates the direction of NCR3LG1 transcription. **B**. Fold change in NCR3LG1 mRNA expression in A549 cells treated as indicated for 30 hours. The graph was prepared based on our published RNA-Seq data [7]. **C**. NCR3LG1 mRNA measured by semi-quantitative real-time polymerase chain reaction in p53-proficient and p53-deficient A549 cells under the same treatment conditions. **D**. Relative NFLA value in U-2 OS cells co-transfected with a reporter vector containing the NCR3LG1 regulatory element and either an empty vector, wild-type p53 expression vector, or mutant p53 expression vector (Val143Ala). **E**. NFLA in U-2 OS cells transfected with the reporter construct and cultured under control conditions or treated with actinomycin D and nutlin-3a (A+N) to activate endogenous p53. Data in panels C-E represent means ± standard deviations from three biological replicates. Statistical significance was determined using Student’s t-test (* *p *< 0.05, ** p < 0.01)

We extended this analysis to another gene, *NCR3LG1*, which also contains ChIP-Seq p53 peak but is located within putative intronic enhancer regions (Fig. 5A). ActD and Nut3a collaborate in *NCR3LG1* activation (Fig. 5B), while CPT is a less potent inducer. Semi-quantitative real-time polymerase chain reaction (RT-PCR) confirmed that A + N induces the gene in a p53-dependent manner (Fig. 5C).

A putative enhancer of *NCR3LG1* is activated by both ectopically expressed and endogenous p53 (Fig. 5D, E). However, the activation of the *NCR3LG1* enhancer by endogenous p53 was modest (~ 3-fold), so we did not compare it in p53-proficient vs. deficient cells. Our transcriptomic data show that expression of *NCR3LG1* is strongly upregulated in A375 cells [7], which is consistent with the hypothesis that p53 activates this gene in a cell-specific manner, in line with its relatively low p53 expression score [22]. Taken together, our findings show that treatment with p53-activating agents upregulates two NK cell-activating ligands, *SLAMF7* and *NCR3LG1*. Therefore, we next investigated whether p53 activation sensitizes cancer cells to NK cell-mediated cytotoxicity.

### Actinomycin D and nutlin-3a sensitize p53-proficient cells to NK-92 cell-mediated killing

NK-92 is a commercially available cell line derived from lymphoma that possesses the functional properties of NK cells. It has been utilized in experimental anticancer therapies, including in vivo human studies [23]. We employed NK-92 cells to determine whether pre-treatment of cancer cells with A + N sensitizes them to NK-mediated cytotoxicity.

**Fig. 6:**
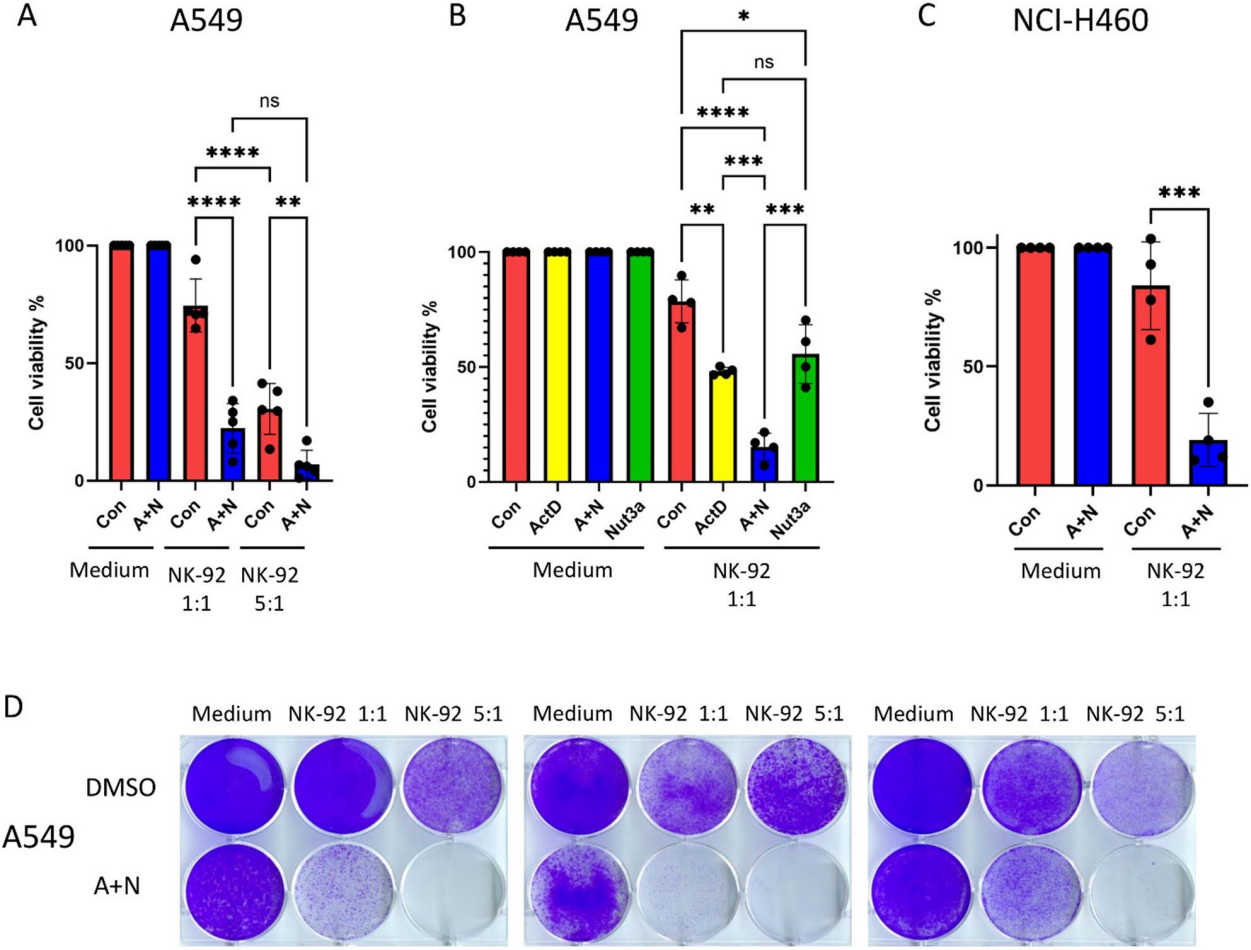
Co-treatment with actinomycin D and nutlin-3a sensitizes cancer cells to killing by NK-92 cells. **A**. Viability of A549 cells measured by MTS assay. Cells were either mock-treated (Con) or treated with actinomycin D (ActD) and nutlin-3a (Nut3a) combined (A+N) for 48 hours, then trypsinized, counted, and incubated for 24 hours in RPMI1640 medium alone or in co-culture with NK-92 cells at two effector-to-target (E: T) ratios (NK-92: A549 – 1:1 and 5:1) for 24 hours. After co-incubation, the medium was replaced with DMEM, and the A549 cells were allowed to recover for 72 hours before assessing metabolic activity via the MTS assay. Five biological replicates were performed. Viability of mock-control cells and drug-treated cells without NK-92 exposure was set to 100%. Statistical analysis: ordinary one-way ANOVA and multiple comparisons test (** *p* < 0.01, **** *p* < 0.0001). **B**. MTS assay of A549 cells pre-treated for 48 hours with ActD, Nut3a, or A+N, followed by 24 hours of co-incubation with NK-92 cells. Viability was assessed as in **A**. Four biological replicates were analyzed. Statistical analysis: ordinary one-way ANOVA and multiple comparisons test (* *p* < 0.05, ** *p* < 0.01, *** *p* < 0.001, **** *p *< 0.0001). **C**. Viability of NCI-H460 cells pre-exposed to A+N or Con for 48 hours, followed by 24 hours of co-incubation with the NK-92 cell line at a 1:1 ratio. Viability was assessed as in **A**. Statistical significance: unpaired, two-tailed t-test (*** *p* < 0.001). **D**. Macroscopic visualization of A549 cells surviving NK-92-mediated killing. Cells were pre-treated for 48 hours with A+N or Con, then seeded on a 6-well plate with or without NK-92 cells at E: T ratios of 1:1 or 5:1 (NK-92:A549). After a 24-hour co-incubation, the RPMI medium for NK-92 cells was replaced with fresh DMEM, and the A549 cells were allowed to recover for 5-7 days. Remaining adherent cells were fixed with methanol and stained with 0.01% crystal violet. Representative results from three biological replicates are shown

First, we pre-treated A549 cells with A + N for 48 h; control cells were mock-treated. These cells were then incubated either in fresh medium or co-cultured with NK-92 cells at effector-to-target (E: T) ratios of 1:1 and 5:1 (Fig. 6A). Note that, in graphs 6A, B, and C, we normalized the viability of cells treated with drugs to 100% in order to illustrate only the influence of NK-92 cells. As will be shown later, the viability of cells exposed to A + N, as measured by MTS assays, was reduced to 25% of the control, primarily due to cell cycle inhibition [16]. As expected, increasing the number of NK-92 cells led to a significant reduction in the viability of control A549 cells. Notably, A + N pre-treatment further sensitized the cells to NK-mediated killing. Under 1:1 conditions, control cell viability dropped to 74%, whereas A + N-treated cells decreased to 22%. A similar trend was observed at a 5:1 ratio. However, most control cells were already eliminated by NK-92 cells alone, and the A + N pre-treatment further reduced viability (Fig. 6A). These results suggest that a 1:1 ratio is a suitable and physiologically relevant ratio for subsequent experiments.

Next, we examined how ActD and Nut3a act individually and in combination to sensitize cancer cells to elimination by NK-92 cells. A549 cells were pre-treated with each compound alone or in combination, followed by co-incubation with NK-92 cells. Both compounds sensitized cancer cells to some extent, but the A + N combination yielded the most substantial effect (Fig. 6B). We then tested this sensitization effect in another p53 wild-type cell line, NCI-H460, and observed a similar pattern (Fig. 6C).

To observe the long-term effect and validate the findings with an alternative viability assay, we employed cell staining with crystal violet (Fig. 6D). A549 cells (mock- or A + N-treated) were co-cultured with NK-92 cells for 24 h, and after the removal of NK-92 cells, the adherent A549 cells were allowed to regrow in fresh medium for 5–7 days (three biological replicates were performed). The cells in the control wells formed a dense monolayer. Although initially affected by exposure to A + N, the cancer cells that survived also recovered (Fig. 6D). However, almost no viable cells remained after pre-treatment with A + N and subsequent co-incubation with NK-92 at a 5:1 ratio, indicating that A + N pre-treatment dramatically increases cancer cell vulnerability to NK cells.

**Fig. 7:**
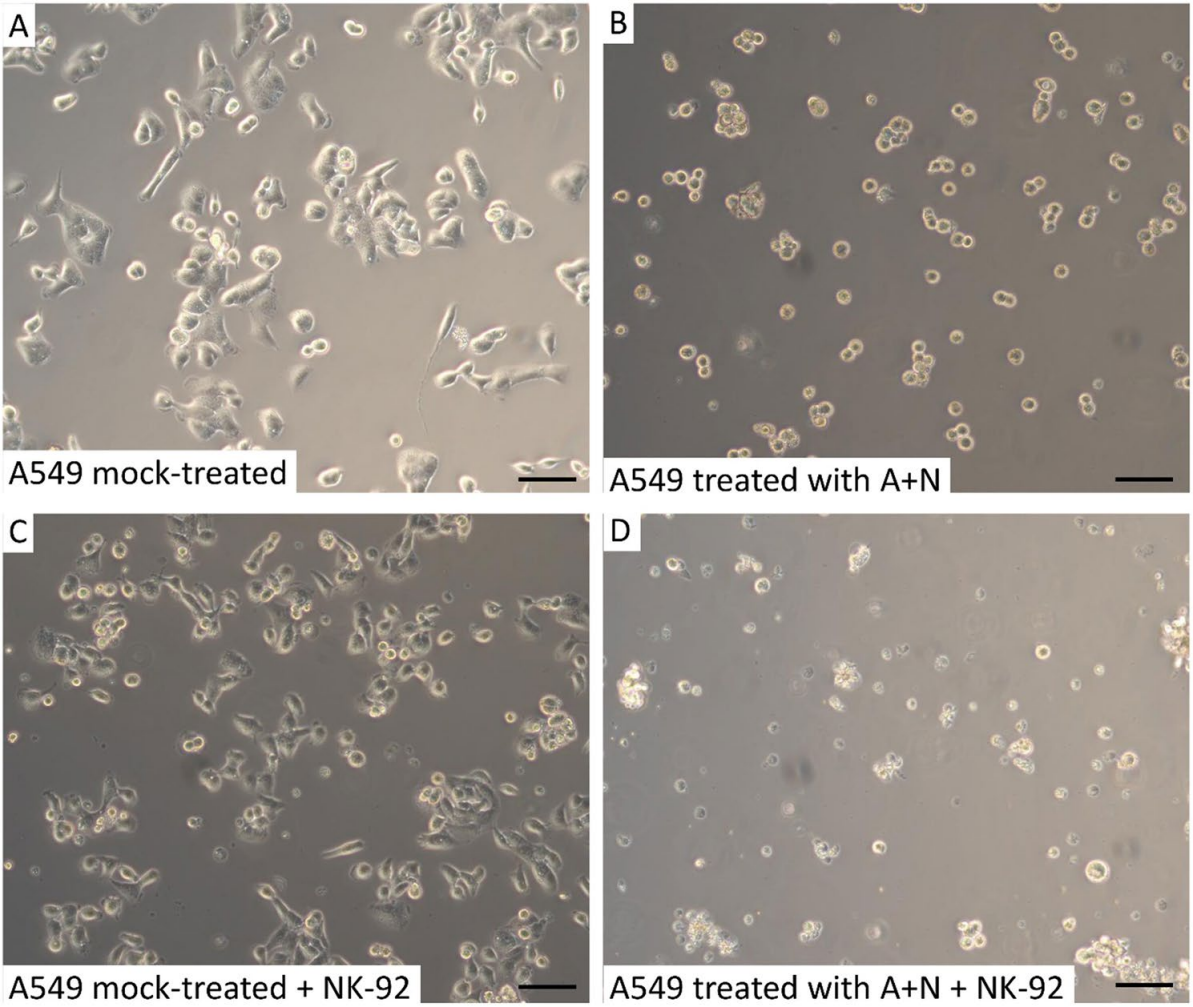
Morphological changes of A549 cells pre-exposed to actinomycin D and nutlin-3a and co-incubated with NK-92 cells suggest extensive elimination of cancer cells. A549 cells were either mock-treated or exposed to actinomycin D and nutlin-3a (A+N) for 48 hours. Subsequently, the A549 cells were trypsinized, counted, and incubated in culture dishes with either blank medium (**A**, **B**) or NK-92 cells at a 1:5 ratio (A549:NK-92) for 24 hours (**C**, **D**). Representative fragments of growth areas were photographed. Panel **A** shows typical morphology of A549 cells growing in control conditions. Treatment with the A+N mixture induces morphological changes, resulting in a more rounded cell shape (**B**). Scale bar = 80 µm

To visualize the cells after 24-hour co-incubation, we made microscopic observations (Fig. 7). The A549 cells, which were neither exposed to A + N nor co-incubated with NK-92 cells, showed typical epithelial-like morphology and were firmly attached to the bottom of culture dish, with no cell debris visible (Fig. 7A). The cells exposed to A + N without NK-92 co-culture demonstrated altered morphology, with a rounded shape typical of A549 cells exposed to this drug combination [16], and barely noticeable cell debris (Fig. 7B). The rounded cells are not pre-mitotic (late G2) cells detaching from the surface because this morphology is visible in almost all cells, while cell cycle distribution analysis show that approximately half of this cell population has G1 content of DNA [16]. The A549 cells growing under control conditions and co-incubated with NK-92 cells were attached to the medium with epithelial-like morphology, but extensive cell debris was also observed (Fig. 7C). Finally, the most conspicuous feature of the images captured from the culture dish with A549 cells exposed to A + N and co-incubated with NK-92 cells was ubiquitous cell debris (Fig. 7D) and large aggregates of NK-92 cells. These large clusters of cells did not form when NK-92 were co-incubated with untreated A549 cells, and probably formed around DNA molecules leaked from dying A549 cells. It is a well-known phenomenon that DNA released from dying cells acts as a “glue” for cell aggregates [24]. Thus, the cultures of A549 cells pre-exposed to A + N and co-incubated with NK-92 cells at a 1:5 ratio displayed several morphological signs of cell death, including prevalent cell debris, scarcity of cells attached to the culture dish, and large aggregates of NK-92 cells, which are readily observable even with the naked eye.

**Fig. 8:**
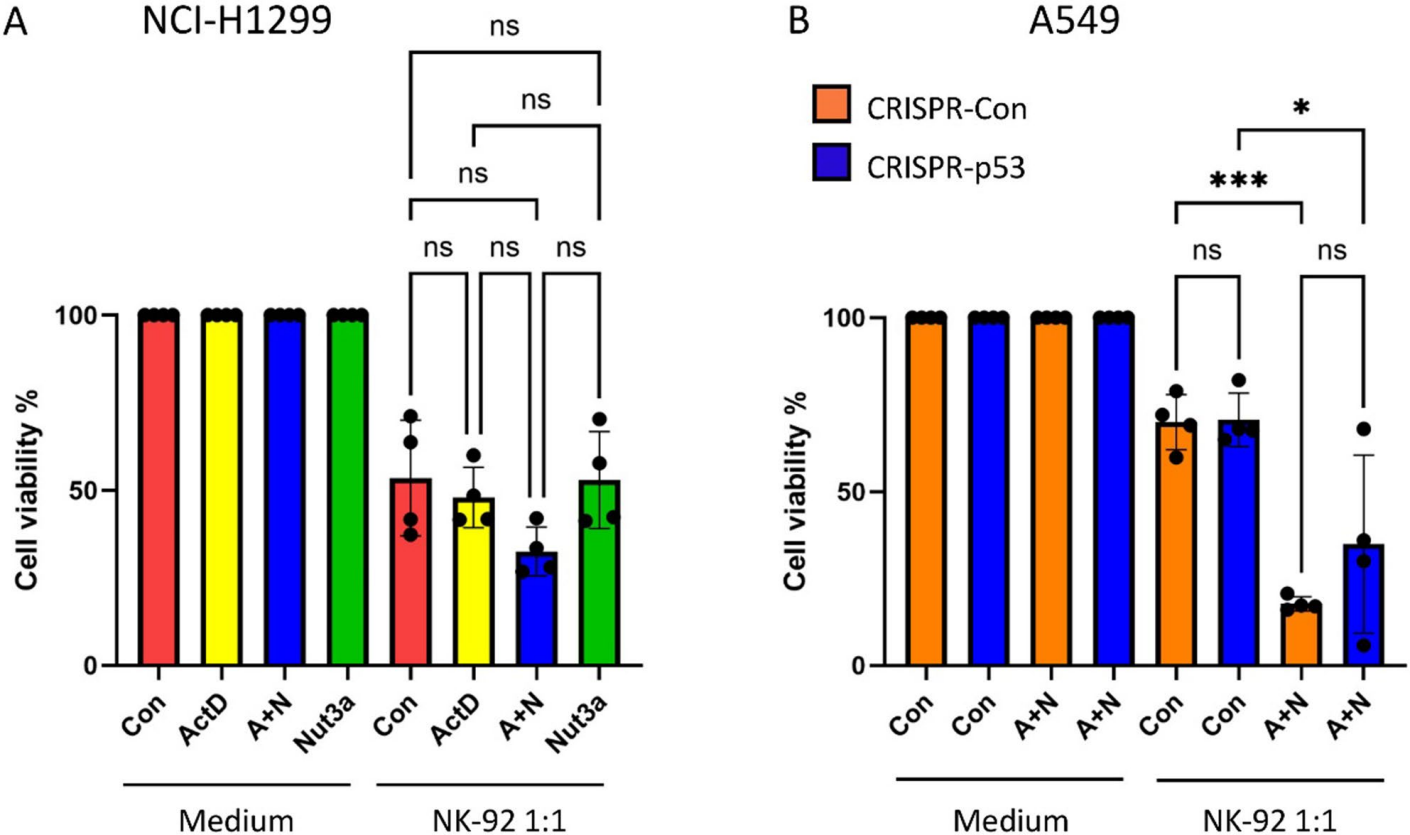
The p53 protein may contribute to the sensitization of cancer cells to natural killer cell-mediated cytotoxicity. **A**. Results of MTS assays performed on NCI-H1299 cells, as described in Fig. 6B. Statistical significance was determined using ordinary one-way ANOVA and multiple comparisons test (ns – non-significant). **B**. Results of MTS assays performed on p53-proficient (CRISPR-Con) and p53-deficient (CRISPR-p53) A549 cells. Cells were mock-treated (Con) or exposed to actinomycin D (ActD) and nutlin-3a (Nut3a) in combination (A+N) for 48 hours, followed by 24 hours of incubation either in medium alone or with NK-92 cells at a 1:1 effector-to-target ratio. After co-incubation, the medium was replaced, and the A549 cells were allowed to recover for 72 hours. Four biological replicates were performed. Statistical significance was calculated using ordinary one-way ANOVA and multiple comparisons test (* *p* < 0.05, *** *p* < 0.001, ns – non-significant)

To assess the role of p53 in this sensitization, we repeated the experiment using p53-null NCI-H1299 cells. These cells were more susceptible to NK-92 elimination at the baseline than A549 cells. However, A + N or single-drug treatments did not significantly enhance this effect (Fig. 8A). For a more direct comparison, we used p53-deficient A549 cells generated via CRISPR and compared them with isogenic p53-proficient controls (Fig. 8B). Fewer p53-deficient cells were killed after A + N pre-treatment and NK-92 exposure, although this difference did not reach statistical significance.

These results demonstrate that cancer cells pre-treated with A + N are almost eliminated by NK-92 cells, suggesting that A + N treatment effectively primes a tumor cell for recognition and eradication by innate immune effectors. Together, these findings indicate that A + N enhances the susceptibility of cancer cells to NK-mediated killing, suggesting that p53 contributes to this sensitizing effect.

### Actinomycin D and nutlin-3a sensitize cancer cells to the cytotoxic activity of primary natural killer cells isolated directly from the blood of the donors

**Fig. 9:**
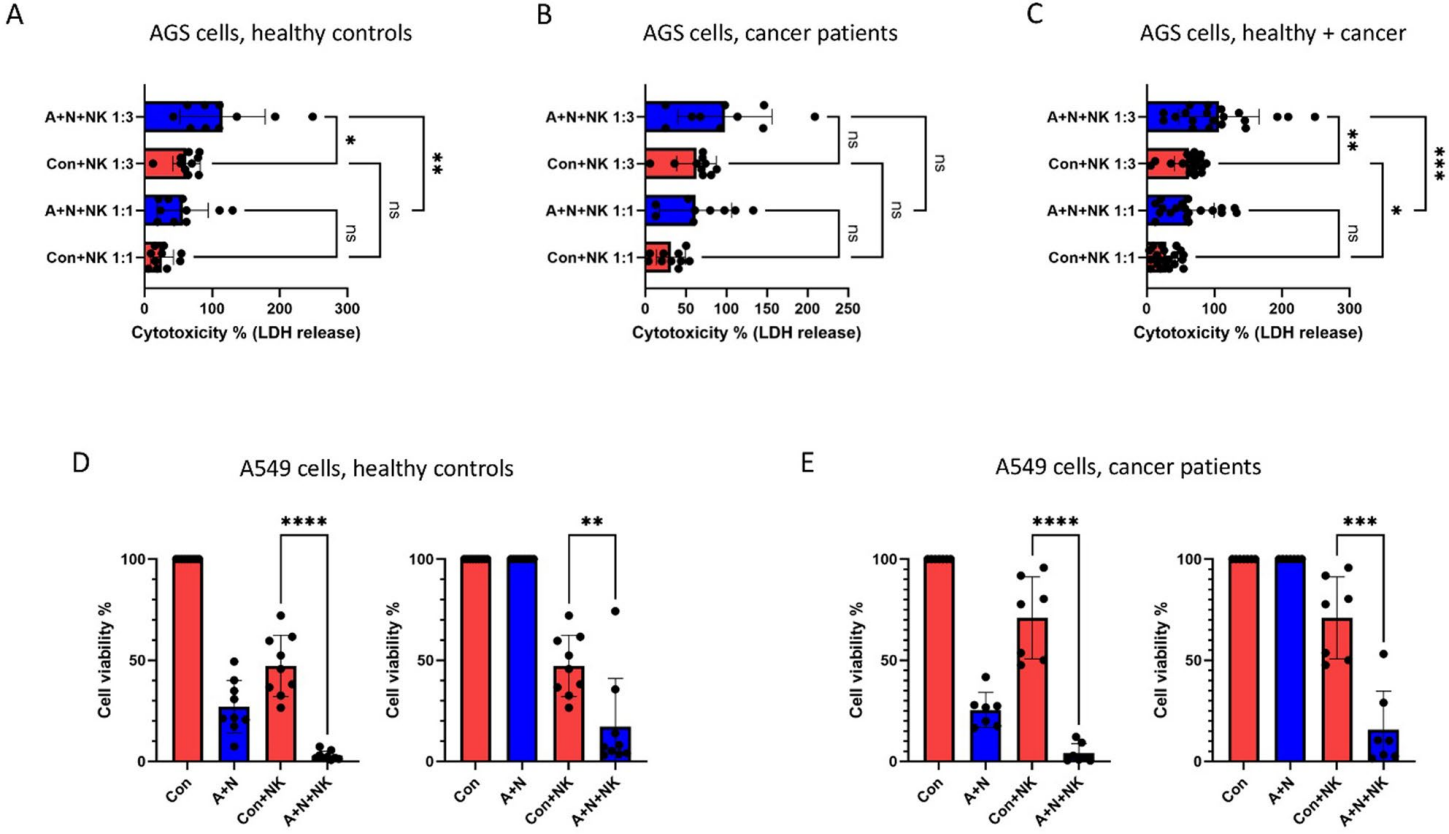
Co-treatment with actinomycin D and nutlin-3a sensitizes cancer cells to killing by primary natural killer cells isolated from healthy donors or cancer patients. **A**. **B**. Cytotoxicity assessed using the lactate dehydrogenase (LDH) cytotoxicity assay on AGS cancer cells cultured under control conditions (Con) or pre-treated with ActD and Nut3a combined (A+N) for 48 hours, followed by 24 hours of co-incubation with natural killer (NK) cells isolated from 10 healthy controls (**A**) or 10 cancer patients **B**. **C**. Combined analysis of the data from panels A and B. Statistical significance was determined using ordinary one-way ANOVA and Šidák’s multiple comparison test (* p<0.05, ** p<0.01, *** p<0.001, ns – non-significant). **D** and **E**. The results of MTS assays for A549 cells that were Con-treated or exposed to A+N for 48 hours. After treatment, cells were trypsinized, counted, and co-cultured for 24 hours either in medium alone or with NK cells isolated from healthy controls (**D**) or cancer patients (**E**) at a 1:1 effector-to-target ratio. Following co-incubation, the medium was replaced, and the A549 cells were allowed to recover for 72 hours before viability was assessed using an MTS assay. In the left panel, the viability of untreated cells (no A+N or NK cells) was set to 100%. In the right panels, to highlight the specific effect of NK cells, the viability was normalized to 100% for both control cells and cells exposed to A+N. Statistical significance was calculated using the unpaired t-test with Welch’s correction (** *p* < 0.05, *** *p* < 0.001, **** *p* < 0.0001).

We tested whether primary NK cells isolated from the peripheral blood of healthy donors and gastric cancer patients can more efficiently eliminate cancer cells pre-treated with A + N. As a model, we used the AGS gastric cancer cell line, which was exposed to A + N for 48 h, followed by a 24-hour co-incubation with primary NK cells. Due to the poor adherence of AGS cells to the culture plate after A + N treatment, the MTS assay was not applicable, so we assessed cytotoxicity based on lactate dehydrogenase (LDH) release. The calculation of percentage toxicity accounted for the cytotoxic effect of A + N alone. As shown in Fig. 9A, pre-treatment with A + N significantly sensitizes AGS cells to NK-mediated killing at an E: T ratio of 3:1. A similar trend was observed at a 1:1 ratio, though it was not statistically significant. Increasing the E: T ratio from 1:1 to 3:1 clearly enhanced the cytotoxic response in both untreated and treated cells (Fig. 9A). Comparable results were observed when NK cells from cancer patients were used (Fig. 9B), and when the data from both donor groups were pooled, the effect reached higher statistical significance (Fig. 9C).

To confirm these findings in another cancer model, we used A549 non-small lung cancer cells and measured their viability after co-incubation with primary NK cells, following the protocol used in Figs. 6 and 8. Cell viability was assessed by MTS assay - the left panels of Fig. 9D and E show that the viability of A549 cells exposed to A + N drops to approximately 25% of the control, as previously shown, primarily due to cell cycle inhibition [16]. Apoptosis and necrosis are rare in these cells, but they are extremely sensitive to apoptosis induced by FAS ligand [2]. Apparently, A549 cells are intrinsically resistant to direct killing by 48-hour treatment with A + N alone. At a 1:1 E: T ratio, A549 cells pre-treated with A + N were consistently more sensitive to NK-mediated killing. This effect was observed for NK cells derived from both healthy individuals (Fig. 9D) and cancer patients (Fig. 9E). It is plausible that part of the sensitizing effect that we observe results from the increased responsiveness of cancer cells exposed to A + N to apoptosis induced by FAS ligand, which plays a significant role in killing of adherent cancer cells by primary human NK cells [25].

## Discussion

In this study, we demonstrated that two immunity-related genes, *SLAMF7* and *NCR3LG1*, are regulated by p53, likely through p53-binding sites localized within their enhancer regions. Two previous high-throughput studies suggested that *SLAMF7* is a p53-regulated gene [7, 26], but here we show that the SLAMF7 protein is actually expressed in non-immune cells. According to a web-based tool developed by Fisher et al. [22], *SLAMF7* has a moderate “p53 expression score” (15/57), indicating that it is activated by p53, but only in certain cell types or under specific stress conditions. However, our transcriptomic data [7] reveal that *SLAMF7* is upregulated by A + N treatment not only in A549 cells but also in U-2 OS and A375 cell lines, which aligns with our Western blotting data (Fig. 1). Figures 1 and 2, and 3 also show that, in our experimental conditions, CPT is a more potent inducer of SLAMF7 than A + N. Moreover, our experiments demonstrate that CPT activates SLAMF7 in all examined cell lines with wild-type p53.

In some cell types, ActD or Nut3a alone was also sufficient to induce expression of SLAMF7. Notably, these specific modes of p53 activation (A + N or CPT) were not included in the datasets analyzed by Fischer et al. [22], which may explain the relatively low p53 expression score for *SLAMF7*. Our findings are of particular interest because derivatives of CPT (e.g., topotecan, irinotecan) are widely used in cancer therapy. While these drugs primarily act as topoisomerase inhibitors, which damage DNA, it is plausible that part of their therapeutic effect involves upregulation of *SLAMF7* and *NCR3LG1*, leading to increased susceptibility of cancer cells to NK-cell-mediated elimination. A similar mechanism may apply to PTX, which, as shown here for the first time, can induce *SLAMF7* expression independently of p53, although this effect appears to be restricted to a limited range of cell lines.

SLAMF7 is constitutively expressed on the surface of various immune cells, including B cells, T cells, dendritic cells, NK cells, and monocytes, with expression levels varying across cell types (e.g., high in plasma cells and low in resting macrophages) [13]. To our knowledge, this is the first study demonstrating that diverse stress signals can robustly induce *SLAMF7* expression in non-immune cells.

As mentioned earlier, SLAMF7 is an activating receptor expressed on the surface of NK cells. Its function has primarily been studied in hematological cancers, as these cells naturally express high levels of SLAMF7 on their surface [9, 27]. However, it remains unclear whether SLAMF7, acting as a ligand on non-hematological cancer cells, contributes to their destruction by NK cells. This question has not been explored previously, likely because it was not known that anticancer drugs can significantly upregulate SLAMF7 expression in such cells. Experiments using HeLa cells (a cervical cancer cell line), engineered to express SLAMF7 from a retroviral vector, demonstrated that SLAMF7 expression enhances the cytolytic activity of both primary NK cells and the NK-92 cell line [9]. These findings suggest that SLAMF7, when present on the surface of non-hematological tumor cells, can promote their elimination by NK cells.

The upregulation of SLAMF7 by anticancer drugs may carry significant therapeutic implications. SLAMF7 is highly expressed on the surface of MM cells and serves as a target for the monoclonal antibody Elotuzumab, which is used in MM treatment. This raises the question of why MM cells exhibit such high SLAMF7 expression and what the precise therapeutic mechanism of Elotuzumab is. SLAMF7, when overexpressed on MM cells, facilitates their adhesion to bone marrow stromal cells, thereby promoting MM cell proliferation and survival through the activation of signaling pathways involving extracellular signal-regulated kinase 1 and 2 (ERK1/2), signal transducer and activator of transcription 3 (STAT3), and protein kinase B (AKT) proteins [13]. Elotuzumab binding to SLAMF7 on MM cells promotes their destruction through several mechanisms. One is antibody-dependent cellular cytotoxicity (ADCC), mediated by NK cells using their CD16 receptor, which binds Elotuzumab attached to SLAMF7. Similarly, this antibody may trigger MM cell destruction by macrophages through antibody-dependent cellular phagocytosis (ADCP). Moreover, Elotuzumab binds to SLAMF7 on NK cells, enhancing their cytotoxic activity. Finally, Elotuzumab binding to SLAMF7 on MM cells may disrupt their growth-promoting interaction with bone marrow stromal cells (reviewed by Wang et al. [28]). It is plausible that CPT-induced upregulation of SLAMF7 on cancer cells enhances their susceptibility to NK cell-mediated killing. Furthermore, the exciting possibility exists that SLAMF7 upregulation on the surface of cancer cells other than MM may render them suitable targets for Elotuzumab and ADCC.

We observed that a higher molecular weight fragment of SLAMF7 (approximately 50 kDa) is present in the extracellular space of cells with induced expression of this protein. This finding is consistent with the detection of soluble SLAMF7 (sSLAMF7) in the serum of a subset of patients with MM. Previous studies have demonstrated that sSLAMF7 promotes the growth of MM cells through homophilic interaction with membrane-bound SLAMF7, leading to the activation of downstream signaling pathways [29]. Our results suggest that sSLAMF7 may also be present in the plasma of patients with other cancers who are exposed to CPT-derived drugs. In MM, sSLAMF7 has been identified as a predictive biomarker for the response to Elotuzumab therapy [30]. It is plausible that sSLAMF7 is also present in the serum of patients with other cancers treated with anticancer drugs. If confirmed, it may be valuable to assess whether sSLAMF7 levels correlate with clinical outcomes following treatment with PTX, CPT analogs, or other chemotherapeutic agents. Notably, our study is the first to report the approximate molecular weight of sSLAMF7, as previous publications relied exclusively on flow cytometry or enzyme-linked immunosorbent assay (ELISA) data [29, 31].

A recent study identified SLAMF7 expression in breast cancer tumors as a strong prognostic factor [32]. However, this conclusion requires confirmation in an independent study, preferably using a different antibody to evaluate SLAMF7 expression in tissue sections. Another investigation, which employed immunohistochemical methods to assess SLAMF7 expression in ovarian cancers, reported variable expression in the interstitial (non-neoplastic) component of the tumor tissue [33]. This finding aligns with previous observations that SLAMF7 is primarily expressed in immune cells. The authors further showed that higher SLAMF7 expression correlated with a favorable prognosis in ovarian cancer patients and was associated with an increase in T-cell infiltration into tumors.

Lingel et al. reported fascinating observations regarding the role of SLAMF7 in the immune system and its influence on cancers [34]. According to their study, SLAMF7 is rapidly expressed following initial antigen recognition of CD8 + T cells, and its surface presence serves as a marker of their commitment to execute cytotoxic responses. In other words, SLAMF7 facilitates the clustering of T cells around antigen-presenting cells and promotes their clonal expansion. Notably, Lingel et al. [34] demonstrated that SLAMF7 enhances the T-cell response to an antigen, particularly in cases of low-affinity antigens. Based on these findings, it can be hypothesized that SLAMF7, when induced on the surface of cancer cells by various stress-related factors, may facilitate interaction with immune cells, such as adhesion to T cells and NK cells. Our results open a new area of investigation into the potential role of SLAMF7 in the treatment of non-hematological malignancies and suggest the possibility of extending the use of Elotuzumab.

This report clearly demonstrates that ActD and Nut3a, which synergistically activate p53, sensitize various cancer cell lines to killing by both NK-92 cells (used in experimental anticancer therapies) and primary NK cells freshly isolated from blood. Indeed, p53 is likely the mediator of this sensitization through the activation of at least three ligands on the surface of cancer cells that stimulate the NK cell response. These ligands include SLAMF7 and NCR3LG1, investigated in this study, as well as ULBP2, which has been previously studied by others [4, 5]. This finding is consistent with the role of p53 as a tumor suppressor, suggesting that p53 enhances the immune system’s ability to eliminate cancer cells.

Is the activation of these three genes the only mechanism by which p53 promotes immune-mediated killing of cancer cells? Probably not; firstly, it is very likely that additional activating ligands for NK cells present on cancer cells remain to be discovered. There are p53-regulated genes with poorly characterized functions, but with features suggesting their involvement in immune responses. Moreover, some key properties of p53 target genes may only be revealed in vivo. However, even if appropriate mouse models were available, one must consider the significant differences in p53 signaling between mice and humans [35]. The development of complex, multicellular in vitro models using human 3D or organoid cultures is a promising direction for future studies [36]. For now, treatment of cells with A + N (or CPT) provides a valuable tool for investigating the molecular role of SLAMF7 in the immune-mediated killing of non-hematological cancer cells.

The NK-92 cell line is used in experimental anticancer therapy for humans. These cells address the challenges associated with isolating and expanding NK cells from donor or patient blood. To date, more than 100 cancer patients have been treated with NK-92 cells. Since the original cell line was derived from a patient with lymphoma, the cells are irradiated prior to infusion to minimize the risk of uncontrolled proliferation in the recipient. Importantly, after irradiation, NK-92 cells retain their ability to kill cancer cells. According to a review by Klingemann [23], approximately 36% of treated patients experienced some tumor reduction, with only minor side effects. Our results suggest that administering chemotherapy prior to infusion could potentially improve patient outcomes.

The sensitizing effect of chemotherapeutic agents on the killing of cancer cells by NK cells has also been observed by other researchers (reviewed by Zingoni et al. [37]). There is consensus that the mechanism of sensitization involves upregulation of activating ligands and death receptors, as well as downregulation of inhibitory ligands on the surface of cancer cells. However, most of these experiments have been conducted using hematological malignancies as models, with MM being the most extensively studied. Therefore, our experiments using cell lines derived from solid tumors (specifically, lung cancer) add an important element of novelty.

Interestingly, one study involving A549 lung cancer cells and NK-92 cells employed a slightly different approach. In that investigation, NK-92 cells were stimulated with an experimental immunomodulatory agent, while A549 cells were pre-treated with the chemotherapeutic drug 5-fluorouracil before co-culture. The effect of 5-fluorouracil alone on cancer cell killing was small and not statistically significant. Enhanced killing was observed only when both agents were applied together [38].

Our experiments are among the few that clearly demonstrate a synergistic effect of two anticancer agents in sensitizing cancer cells to NK cell-mediated killing. Our data suggest that this sensitization is primarily driven by the strong p53-dependent activation of genes encoding ligands for activating receptors, although p53-independent mechanisms may also contribute. In this study, we focused on the regulation of SLAMF7 expression; however, we also showed that another ligand, NCR3LG1, is upregulated by A + N in a p53-dependent manner. This is a significant observation, as a recently published comprehensive clinical, in silico, in vitro, and in vivo investigation identified NCR3LG1 as the predominant activating ligand responsible for NK cell-mediated cytotoxicity in patients with hematologic cancers (liquid tumors) [11]. A decade earlier, Cao et al. [39] demonstrated that NCR3LG1 is upregulated by cis-Pt, 5-fluorouracil, and irradiation – although they did not investigate the role of p53 in this process. Given that all of these agents are known p53 activators, the hypothesis that p53 regulates *NCR3LG1* and thus plays a key role in sensitizing cancer cells to NK cell killing seems both plausible and important.

## Materials and methods

### Cell culture and treatment

A549 (RRID: CVCL_0023, lung adenocarcinoma), NCI-H292 (RRID: CVCL_0455, lung mucoepidermoid carcinoma), NCI-H1299 cells (RRID: CVCL_0060, large cell carcinoma of the lung) and U-2 OS (RRID: CVCL_0042, osteosarcoma) cell lines (ATCC, Manassas, VA, USA) were cultured in low-glucose DMEM supplemented with 10% fetal bovine serum (FBS; Invitrogen, Carlsbad, CA, USA). A375 cells (RRID: CVCL_0132, melanoma) were cultured in high-glucose DMEM supplemented with 10% FBS. NCI-H460 cells (RRID: CVCL_0459, lung cancer, ATCC) and WM793B cells (RRID: CVCL_8787, melanoma) were cultured in RPMI-1640 supplemented with 2 mM L-glutamine, 4.5 g/L glucose, 1 mM sodium pyruvate, and 10% heat-inactivated FBS. The AGS gastric adenocarcinoma cell line (RRID: CVCL_0139, ATCC) was cultured in McCoy’s 5 A medium with 10% FBS. WM278 cells (RRID: CVCL_6473, melanoma) were cultured in RPMI-1640 with 20% FBS. NK-92 cells (RRID: CVCL_2142, malignant non-Hodgkin lymphoma) were cultured on an RPMI-1640 medium supplemented with 2 mM glutamine, 1mM sodium pyruvate, 20 ng/ml interleukin-2 (IL-2) (Miltenyi Biotech, Bergisch Gladbach, Germany), 12.5% FBS, and 12.5% horse serum (HS) (Biowest, Nuaillé, France). All media were supplemented with a penicillin/streptomycin solution. The cells were incubated at 37 °C and 5% CO_2_ with saturated humidity and were regularly tested for mycoplasma contamination.

Stock solutions of the following chemicals were prepared in DMSO (dimethyl sulfoxide): ActD (10 µM; Sigma-Aldrich, St. Louis, MO, USA), CPT (10 mM; Calbiochem-Merck, Darmstadt, Germany), and Nut3a (10 mM; Selleck Chemicals LLC, Houston, TX, USA). The stock solutions of PTX (7 mM; EBEWE Pharma), Eto (34 mM; EBEWE Pharma), and cisplatinum (3.33 mM; EBEWE Pharma) were formulated by the vendor. The stock solutions were diluted in culture medium to the following concentrations: 5 nM ActD, 5µM Nut3a, 5 µM CPT, 0.08-10 µM PTX (as shown in Results), 15 µM Eto, and 10 µM cisplatinum. The control cells were mock-treated with medium containing DMSO.

### Generation of p53-deficient cells

The generation of p53-deficient A549 and U-2 OS cells using CRISPR/Cas9 technology was previously described by Łasut-Szyszka et al. [6]. The same method was employed to generate the p53-deficient NCI-H460 cells. All p53-deficient cell lines were cultured under the same conditions as their wild-type counterparts.

### Western blotting

The preparation of whole-cell lysates using IP buffer supplemented with protease and phosphatase inhibitors, as well as the preparation of concentrated conditioned medium, was described previously [40]. Aliquots of lysates (35–50 µg) were separated by SDS‒PAGE on 8% or 13% gels and electrotransferred onto PVDF membranes. Prior to incubation with primary antibodies, membranes were blocked for one hour at room temperature (RT) in 5% skimmed milk in phosphate-buffered saline (PBS) containing 0.1% Tween-20.

Antibodies against phospho-Ser37-p53, phospho-Ser15-p53, STING1 and SLAMF7 were purchased from Cell Signaling Technology (Danvers, MA, USA). Antibodies against p53 (DO-1), p21 (F-5), and the loading control, HSC70 (B-6), were obtained from Santa Cruz Biotechnology (Dallas, TX, USA). All primary antibody incubations were carried out overnight at 4 °C in blocking solution. Horse radish peroxidase (HRP)-conjugated secondary antibodies (anti-mouse, anti-rabbit; Cell Signaling Technology) were detected using chemiluminescence with either SuperSignal West Pico or SuperSignal West Femto Chemiluminescent substrate (Thermo Fisher Scientific, Waltham, MA, USA).

### Gene expression analysis by semi-quantitative real-time polymerase chain reaction

After treatment, the cells were harvested by trypsinization, washed with PBS, and then frozen on dry ice, before being stored at -80 °C. Total RNA was isolated using the RNeasy mini kit (Qiagen, Hilden, Germany), and complimentary DNA (cDNA) synthesis was performed with M-MLV reverse transcriptase and random hexamers (Applied Biosystems, Foster City, CA, USA). Gene expression was measured using Real-Time 2x PCR Master Mix SYBR (A&A Biotechnology, Gdynia, Poland). Primer sequences used for semi-quantitative real-time PCR are listed in Supplementary Table 1. Amplification was carried out on a CFX96 Real-Time System (Bio-Rad, Hercules, CA, USA). Each cDNA sample was amplified in triplicate. Relative mRNA quantitation was performed using the ΔΔCT method, with actin, beta (*ACTB)*, or glyceraldehyde-3-phosphate dehydrogenase (*GAPDH*) as reference genes. Means and standard deviations were calculated from three biological replicates.

### Cloning of gene regulatory elements and luciferase reporter assays

Gene regulatory elements of *SLAMF7* and *NCR3LG1* were cloned into the pGL3-Basic reporter vector, which encodes firefly luciferase (Promega, Madison, WI, USA), using the method described previously [40]. First, the fragments were PCR-amplified from genomic DNA isolated from the A549 cell line. The primers contained artificially introduced restriction sites for cloning into compatible sites of the pGL3-Basic vector. Primer sequences are as follows: restriction sites are underlined, and the corresponding enzymes are given in parentheses.

* SLAMF7*:

- 5’-TTTT GAGCTC TCC TGG CTG GCT TAT CAC TG-3’ (SacI)
- 5’-TTTT ACGCGT GCC TAC TCA GGC AGT GTT GTA C-3’ (MluI)

*NCR3LG1*:

- 5’-TTTT GAGCTC TCT GCA CAA CAG CCA GTA CAT C-3’ (SacI)
- 5’-TTTT ACGCGT AGT CTC GTC AAT GCA CCA CAA TG-3’ (MluI).

PCR amplification of *SLAMF7* or *NCR3LG1* fragments was performed using PfuPlus! DNA polymerase mix (EURx, Gdańsk, Poland) to ensure high-fidelity amplification. The amplified fragments were ligated into the SacI and MluI sites of the pGL3-Basic vector. The generated plasmids were sequenced to ensure that the clones contained no mutations.

The luciferase reporter assay was conducted as described previously [40]. Briefly, U-2 OS cells were co-transfected using FuGene6 (Promega, WI, USA) with a combination of the reporter vector - encoding firefly luciferase under the control of the cloned regulatory elements - and expression vector encoding either wild-type p53 (pC53-SN3) or the Val143Ala p53 mutant (pC53-SCX3), a gift from Dr. Bert Vogelstein and Dr. Kenneth W. Kinzler (Johns Hopkins University, Baltimore, MD, USA) [41]. As a negative control, the p53 expression plasmid was replaced by an empty vector. The transfection mixture also contained *Renilla* luciferase thymidine kinase (pRL-TK), encoding *Renilla sp.* luciferase under the control of the herpes simplex virus thymidine kinase (HSV-TK) promoter, used as an internal control.

The next day, cells were washed with culture medium and incubated with fresh medium for an additional 24 h. Subsequently, cells were lysed with passive lysis buffer (PLB) from the Dual Luciferase Reporter Assay system (Promega, WI, USA), and luciferase activities were measured. Firefly luciferase activity was normalized to *Renilla sp.* luciferase activity, yielding NFLA. Each transfection was performed in triplicate in three independent experiments.

To assess the impact of endogenous p53 on the activity of cloned regulatory elements, cells were transfected with the reporter vectors and the pRL-TK control vector. After 24 h, the transfection mixture (FuGENE 6 + DNA) was removed, and the cells were exposed to either control medium or medium containing A + N to activate endogenous p53. After an additional 24 h, luciferase activities were measured, and NFLA was calculated as described above.

### Killing of cancer cells by NK-92 cells

Cancer cells cultured in their respective media on 6-cm culture dishes were either mock-treated or exposed for 48 h to the drugs or their combinations at the concentrations specified above. After treatment, the cells were trypsinized, washed with PBS, and then centrifuged. The resulting cell pellets were resuspended in NK-92 cell medium.

Control or drug-treated cells were then seeded into a 96-well plate, together with NK-92 cells, at the E: T ratios specified in the Results section. Specifically, 3,500 cancer cells were seeded per well in 100 µl of NK-92 medium (RPMI-1640 with supplements). For the control, cancer cells were seeded without NK-92 cells. Each experimental condition was performed in triplicate. Co-incubation lasted for 24 h, after which the medium was removed and replaced with cancer cell culture medium. Surviving adherent cancer cells were allowed to recover for 72 h. Cell metabolic activity was then measured using the MTS assay (CellTiter 96^®^ AQ_ueous_ One Solution Cell Proliferation Assay, cat no. G3582; Promega, Madison, WI, USA). The metabolic activity of cancer cells cultured in the absence of NK-92 cells was set as 100%.

To visualize adherent A549 cells surviving the co-treatment with NK-92 cells (which grow in suspension), A549 cells were mock-treated or treated with A + N for 48 h in DMEM. After treatment, cells were trypsinized, counted, and 100,000 cells were seeded per well in a 6-well plate. NK-92 cells were also counted and added to the same wells at NK-92:A549 ratios of 1:1 and 5:1. Control wells contained A549 cells incubated only with NK-92 medium. Cells were co-incubated in 2 ml of NK-92 medium per well. After 24 h of co-incubation, RPMI-1640 medium was removed, and adherent cells were allowed to recover in fresh DMEM for 5–7 days. Subsequently, the medium was removed, cells were rinsed with PBS, and fixed by incubation with cold methanol (-20 °C) for 10 min. Fixed and dried cells were stained with 0.01% crystal violet for 2 min, washed with distilled water, air-dried, and scanned.

### Live-cell imaging

A549 cells were mock-treated or exposed to A + N for 48 h in DMEM. After treatment, cells were trypsinized, counted, and 50,000 cells were seeded per well in a standard 12-well plate. NK-92 cells were also counted and added to the same wells at a 5:1 ratio (NK-92: A549). Control wells contained A549 cells (either mock-treated or exposed to A + N) incubated only with RPMI medium for NK-92 cells. After 24 h of co-incubation, the representative fragments of growth areas were photographed. The images were acquired using an Axiovert 5 inverted microscope equipped with standard software (ZEN lite; Carl Zeiss, Jena, Germany).

### Isolation of primary human natural killer cells and testing of their anticancer properties

Primary NK cells were isolated from 12 ml of whole blood either from healthy donors or from gastric cancer patients treated at the Maria Skłodowska-Curie National Research Institute of Oncology in Gliwice (NK cells were collected from patients prior to any treatment). The isolation was performed using the Human NK Cell Isolation Kit (Miltenyi Biotec, Bergisch Gladbach, Germany) according to the manufacturer’s instructions.

Following isolation, the cells were cultured for 24 h under standard conditions in RPMI-1640 medium supplemented with 12.5% FBS, 12.5% HS, 20 ng/ml IL-2, and 20 ng/ml IL-15 (Miltenyi Biotec, Bergisch Gladbach, Germany).

AGS tumor cells were either mock-treated or exposed to A + N for 48 h. After treatment, the cancer cells were trypsinized and counted. Treated or control AGS cells were then co-incubated with primary NK cells in 1000 µl of serum-free RPMI-1640 medium in the wells of a 24-well plate for 24 h. The cytotoxic effects of NK cells were assessed by measuring the LDH released into the culture medium from damaged or dying cells. Serum-free medium was used to avoid background LDH originating from serum.

After 24 h of co-incubation, 100 µl of medium was collected and subjected to centrifugation at 2000 rpm for 2 min at RT to remove cells or debris. Then, 10 µl of the cleared supernatant was mixed with 40 µl of Storage Buffer (200 mM Tris-HCl, pH 7.3, 10% glycerol, 1% BSA), and the resulting mixture was used for LDH measurement using an LDH-Glo™ Cytotoxicity Assay kit from Promega. The percentage of cytotoxicity was calculated for each E: T ratio (NK: AGS) using the following formula:

$$\%\;cytotoxicity=\frac{\mathrm{E}\mathrm{x}\mathrm{p}\mathrm{e}\mathrm{r}\mathrm{i}\mathrm{m}\mathrm{e}\mathrm{n}\mathrm{t}\mathrm{a}\mathrm{l}\;\mathrm{L}\mathrm{D}\mathrm{H}-\mathrm{E}\mathrm{f}\mathrm{f}\mathrm{e}\mathrm{c}\mathrm{t}\mathrm{o}\mathrm{r}\;\mathrm{s}\mathrm{p}\mathrm{o}\mathrm{n}\mathrm{t}\mathrm{a}\mathrm{n}\mathrm{e}\mathrm{o}\mathrm{u}\mathrm{s}\;\mathrm{L}\mathrm{D}\mathrm{H}-\mathrm{T}\mathrm{a}\mathrm{r}\mathrm{g}\mathrm{e}\mathrm{t}\;\mathrm{s}\mathrm{p}\mathrm{o}\mathrm{n}\mathrm{t}\mathrm{a}\mathrm{n}\mathrm{e}\mathrm{u}\mathrm{o}\mathrm{s}\;\mathrm{L}\mathrm{D}\mathrm{H}}{\mathrm{T}\mathrm{a}\mathrm{r}\mathrm{g}\mathrm{e}\mathrm{t}\;\mathrm{m}\mathrm{a}\mathrm{x}\mathrm{i}\mathrm{m}\mathrm{u}\mathrm{m}\;\mathrm{L}\mathrm{D}\mathrm{H}-\mathrm{T}\mathrm{a}\mathrm{r}\mathrm{g}\mathrm{e}\mathrm{t}\;\mathrm{s}\mathrm{p}\mathrm{o}\mathrm{n}\mathrm{t}\mathrm{a}\mathrm{n}\mathrm{e}\mathrm{o}\mathrm{u}\mathrm{s}\;\mathrm{L}\mathrm{D}\mathrm{H}}x100$$

To calculate the percent cytotoxicity of AGS cells pre-exposed to A + N and co-incubated with NK cells at a 1:1 ratio, we used the following LDH activities: Experimental LDH – the value from AGS cells pre-exposed to A + N and incubated with NK cells; Effector spontaneous LDH from the same number of NK cells cultured alone; Target spontaneous LDH from AGS cells treated with A + N but without NK cells; Target maximum LDH from AGS cells treated with A + N and incubated with 1% Triton X-100 for 30 min to induce complete cell lysis.

To assess the effect of primary NK cells on A549 cell viability using the MTS assay, we followed the procedure described in the previous paragraph, substituting NK-92 cells with primary NK cells.

### Statistical analysis

All tests were performed using GraphPad Prism version 10.2.2 for Windows (GraphPad Software, Boston, MA, USA; www.graphpad.com). The types of tests used are mentioned in the relevant figure captions.

## Supplementary Information

Supplementary Material 1.

## Data Availability

No datasets were generated or analyzed during the current study.

## References

[1] Liu Y, Stockwell BR, Jiang X, Gu W. p53-regulated non-apoptotic cell death pathways and their relevance in cancer and other diseases. Nat Rev Mol Cell Biol. 2025 Apr 9. 10.1038/s41580-025-00842-3. Epub ahead of print. PMID: 40204927. 10.1038/s41580-025-00842-3 PMC13014097 40204927

[2] Łasut-Szyszka B, Gdowicz-Kłosok A, Krześniak M, Głowala-Kosińska M, Będzińska A, Rusin M. Strong activation of p53 by actinomycin D and nutlin-3a overcomes the resistance of cancer cells to the pro-apoptotic activity of the FAS ligand. Apoptosis. 2024;29(9–10):1515–28. 10.1007/s10495-024-02000-0. Epub 2024 Jul 28. PMID: 39068622; PMCID: PMC11416401. 39068622 10.1007/s10495-024-02000-0 PMC11416401

[3] Álvarez-Carrasco P, Maldonado-Bernal C. The innate defenders: a review of natural killer cell immunotherapies in cancer. Front Immunol. 2024;15:1482807. 10.3389/fimmu.2024.1482807. PMID: 39763648; PMCID: PMC11700995. 10.3389/fimmu.2024.1482807 PMC11700995 39763648

[4] Li H, Lakshmikanth T, Garofalo C, Enge M, Spinnler C, Anichini A, Szekely L, Kärre K, Carbone E, Selivanova G. Pharmacological activation of p53 triggers anticancer innate immune response through induction of ULBP2. Cell Cycle. 2011;10(19):3346-58. 10.4161/cc.10.19.17630. Epub 2011 Oct 1. PMID: 21941086. 10.4161/cc.10.19.17630 21941086

[5] Textor S, Fiegler N, Arnold A, Porgador A, Hofmann TG, Cerwenka A. Human NK cells are alerted to induction of p53 in cancer cells by upregulation of the NKG2D ligands ULBP1 and ULBP2. Cancer Res. 2011;71(18):5998–6009. 10.1158/0008-5472.CAN-10-3211. Epub 2011 Jul 15. PMID: 21764762. 21764762 10.1158/0008-5472.CAN-10-3211

[6] Łasut-Szyszka B, Małachowska B, Gdowicz-Kłosok A, Krześniak M, Głowala-Kosińska M, Zajkowicz A, Rusin M. Transcriptome Analysis of Cells Exposed to Actinomycin D and Nutlin-3a Reveals New Candidate p53-Target Genes and Indicates That CHIR-98014 Is an Important Inhibitor of p53 Activity. Int J Mol Sci. 2021;22(20):11072. 10.3390/ijms222011072. PMID: 34681730; PMCID: PMC8538697. 34681730 10.3390/ijms222011072 PMC8538697

[7] Łasut-Szyszka B, Gdowicz-Kłosok A, Małachowska B, Krześniak M, Będzińska A, Gawin M, Pietrowska M, Rusin M. Transcriptomic and proteomic study of cancer cell lines exposed to actinomycin D and nutlin-3a reveals numerous, novel candidates for p53-regulated genes. Chem Biol Interact. 2024;392:110946. Epub 2024 Mar 7. PMID: 38460933. 38460933 10.1016/j.cbi.2024.110946

[8] Kumaresan PR, Lai WC, Chuang SS, Bennett M, Mathew PA. CS1, a novel member of the CD2 family, is homophilic and regulates NK cell function. Mol Immunol. 2002;39(1–2):1–8. 10.1016/s0161-5890(02)00094-9. PMID: 12213321. 10.1016/s0161-5890(02)00094-9 12213321

[9] Gutierrez-Guerrero A, Mancilla-Herrera I, Maravillas-Montero JL, Martinez-Duncker I, Veillette A, Cruz-Munoz ME. SLAMF7 selectively favors degranulation to promote cytotoxicity in human NK cells. Eur J Immunol. 2022;52(1):62–74. 10.1002/eji.202149406. Epub 2021 Nov 14. PMID: 34693521. 34693521 10.1002/eji.202149406

[10] Brandt CS, Baratin M, Yi EC, Kennedy J, Gao Z, Fox B, Haldeman B, Ostrander CD, Kaifu T, Chabannon C, Moretta A, West R, Xu W, Vivier E, Levin SD. The B7 family member B7-H6 is a tumor cell ligand for the activating natural killer cell receptor NKp30 in humans. J Exp Med. 2009;206(7):1495–503. 10.1084/jem.20090681. Epub 2009 Jun 15. PMID: 19528259; PMCID: PMC2715080. 19528259 10.1084/jem.20090681 PMC2715080

[11] Lee S, Kim JH, Jang IH, Jo S, Lee SY, Oh SC, Kim SM, Kong L, Ko J, Kim TD. Harnessing B7-H6 for Anticancer Immunotherapy: Expression, Pathways, and Therapeutic Strategies. Int J Mol Sci. 2024;25(19):10326. 10.3390/ijms251910326. PMID: 39408655; PMCID: PMC11476788. 39408655 10.3390/ijms251910326 PMC11476788

[12] Wang SH, Chou WC, Huang HC, Lee TA, Hsiao TC, Wang LH, Huang KB, Kuo CT, Chao CH, Chang SJ, Hsu JM, Weng J, Ren N, Li FA, Lai YJ, Zhou C, Hung MC, Li CW. Deglycosylation of SLAMF7 in breast cancers enhances phagocytosis. Am J Cancer Res. 2022;12(10):4721–36. PMID: 36381324; PMCID: PMC9641385. 36381324 PMC9641385

[13] Chu E, Wu J, Kang SS, Kang Y. SLAMF7 as a Promising Immunotherapeutic Target in Multiple Myeloma Treatments. Curr Oncol. 2023;30(9):7891–903. 10.3390/curroncol30090573. PMID: 37754488; PMCID: PMC10529721. 37754488 10.3390/curroncol30090573 PMC10529721

[14] Tai YT, Dillon M, Song W, Leiba M, Li XF, Burger P, Lee AI, Podar K, Hideshima T, Rice AG, van Abbema A, Jesaitis L, Caras I, Law D, Weller E, Xie W, Richardson P, Munshi NC, Mathiot C, Avet-Loiseau H, Afar DE, Anderson KC. Anti-CS1 humanized monoclonal antibody HuLuc63 inhibits myeloma cell adhesion and induces antibody-dependent cellular cytotoxicity in the bone marrow milieu. Blood. 2008;112(4):1329–37. 10.1182/blood-2007-08-107292. Epub 2007 Sep 28. PMID: 17906076; PMCID: PMC2515112. 17906076 10.1182/blood-2007-08-107292 PMC2515112

[15] Postelnek J, Neely RJ, Robbins MD, Gleason CR, Peterson JE, Piccoli SP. Development and Validation of Electrochemiluminescence Assays to Measure Free and Total sSLAMF7 in Human Serum in the Absence and Presence of Elotuzumab. AAPS J. 2016;18(4):989–99. 10.1208/s12248-016-9912-3. Epub 2016 Apr 26. PMID: 27116021. 27116021 10.1208/s12248-016-9912-3

[16] Zajkowicz A, Gdowicz-Kłosok A, Krześniak M, Ścieglińska D, Rusin M. Actinomycin D and nutlin-3a synergistically promote phosphorylation of p53 on serine 46 in cancer cell lines of different origin. Cell Signal. 2015;27(9):1677–87. 10.1016/j.cellsig.2015.05.005. Epub 2015 May 16. PMID: 25989210. 25989210 10.1016/j.cellsig.2015.05.005

[17] Oki S, Ohta T, Shioi G, Hatanaka H, Ogasawara O, Okuda Y, Kawaji H, Nakaki R, Sese J, Meno C. ChIP-Atlas: a data-mining suite powered by full integration of public ChIP-seq data. EMBO Rep. 2018 Dec;19(12):e46255. doi: 10.15252/embr.201846255. Epub 2018 Nov 9. PMID: 30413482; PMCID: PMC6280645. 10.15252/embr.201846255 PMC6280645 30413482

[18] Schlereth K, Heyl C, Krampitz AM, Mernberger M, Finkernagel F, Scharfe M, Jarek M, Leich E, Rosenwald A, Stiewe T. Characterization of the p53 cistrome–DNA binding cooperativity dissects p53’s tumor suppressor functions. PLoS Genet. 2013;9(8):e1003726. 10.1371/journal.pgen.1003726. Epub 2013 Aug 15. PMID: 23966881; PMCID: PMC3744428. 23966881 10.1371/journal.pgen.1003726 PMC3744428

[19] Tebaldi T, Zaccara S, Alessandrini F, Bisio A, Ciribilli Y, Inga A. Whole-genome cartography of p53 response elements ranked on transactivation potential. BMC Genomics. 2015;16(1):464. 10.1186/s12864-015-1643-9. PMID: 26081755; PMCID: PMC4470028. 26081755 10.1186/s12864-015-1643-9 PMC4470028

[20] Kim TK, Hemberg M, Gray JM, Costa AM, Bear DM, Wu J, Harmin DA, Laptewicz M, Barbara-Haley K, Kuersten S, Markenscoff-Papadimitriou E, Kuhl D, Bito H, Worley PF, Kreiman G, Greenberg ME. Widespread transcription at neuronal activity-regulated enhancers. Nature. 2010;465(7295):182–7. 10.1038/nature09033. Epub 2010 Apr 14. PMID: 20393465; PMCID: PMC3020079. 20393465 10.1038/nature09033 PMC3020079

[21] Melo CA, Drost J, Wijchers PJ, van de Werken H, de Wit E, Oude Vrielink JA, Elkon R, Melo SA, Léveillé N, Kalluri R, de Laat W, Agami R. eRNAs are required for p53-dependent enhancer activity and gene transcription. Mol Cell. 2013;49(3):524–35. Epub 2012 Dec 27. PMID: 23273978. 23273978 10.1016/j.molcel.2012.11.021

[22] Fischer M, Schwarz R, Riege K, DeCaprio JA, Hoffmann S. TargetGeneReg 2.0: a comprehensive web-atlas for p53, p63, and cell cycle-dependent gene regulation. NAR Cancer. 2022;4(1):zcac009. 10.1093/narcan/zcac009. PMID: 35350773; PMCID: PMC8946727. 35350773 10.1093/narcan/zcac009 PMC8946727

[23] Klingemann H. The NK-92 cell line-30 years later: its impact on natural killer cell research and treatment of cancer. Cytotherapy. 2023;25(5):451–7. 10.1016/j.jcyt.2022.12.003. Epub 2023 Jan 6. PMID: 36610812. 36610812 10.1016/j.jcyt.2022.12.003

[24] Renner WA, Jordan M, Eppenberger HM, Leist C. Cell-cell adhesion and aggregation: Influence on the growth behavior of CHO cells. Biotechnol Bioeng. 1993;41(2):188 – 93. 10.1002/bit.260410204. PMID: 18609537. 10.1002/bit.260410204 18609537

[25] Zhu Y, Huang B, Shi J. Fas ligand and lytic granule differentially control cytotoxic dynamics of natural killer cell against cancer target. Oncotarget. 2016;7(30):47163–47172. 10.18632/oncotarget.9980. PMID: 27323411. 10.18632/oncotarget.9980 PMC5216932 27323411

[26] Zhou H, Cui X, Yuan H, Zhang B, Meng C, Zhao D. Effects of distinct drugs on gene transcription in an osteosarcoma cell line. Oncol Lett. 2017;14(4):4694–700. 10.3892/ol.2017.6767. Epub 2017 Aug 18. PMID: 29085469; PMCID: PMC5649527. 29085469 10.3892/ol.2017.6767 PMC5649527

[27] Altalbawy FMA, Babamuradova Z, Baldaniya L, Singh A, Singh KU, Ballal S, Sabarivani A, Sead FF, Alam R, Alshahrani MY. The multifaceted role of CS1 (SLAMF7) in immunoregulation: Implications for cancer therapy and autoimmune disorders. Exp Cell Res. 2025;447(1):114516. Epub 2025 Mar 10. PMID: 40073958. 40073958 10.1016/j.yexcr.2025.114516

[28] Wang Y, Sanchez L, Siegel DS, Wang ML. Elotuzumab for the treatment of multiple myeloma. J Hematol Oncol. 2016;9(1):55. 10.1186/s13045-016-0284-z. PMID: 27417553; PMCID: PMC4946088. 27417553 10.1186/s13045-016-0284-z PMC4946088

[29] Kikuchi J, Hori M, Iha H, Toyama-Sorimachi N, Hagiwara S, Kuroda Y, Koyama D, Izumi T, Yasui H, Suzuki A, Furukawa Y. Soluble SLAMF7 promotes the growth of myeloma cells via homophilic interaction with surface SLAMF7. Leukemia. 2020;34(1):180–95. 10.1038/s41375-019-0525-6. Epub 2019 Jul 29. PMID: 31358854. 31358854 10.1038/s41375-019-0525-6

[30] Suzuki A, Kakugawa S, Miyoshi M, Hori M, Suzuki K, Furukawa Y, Ohta K. Soluble SLAMF7 is a predictive biomarker for elotuzumab therapy. Leukemia. 2020;34(11):3088–90. 10.1038/s41375-020-0860-7. Epub 2020 May 12. PMID: 32398792. 32398792 10.1038/s41375-020-0860-7

[31] Ishibashi M, Soeda S, Sasaki M, Handa H, Imai Y, Tanaka N, Tanosaki S, Ito S, Odajima T, Sugimori H, Asayama T, Sunakawa M, Kaito Y, Kinoshita R, Kuribayashi Y, Onodera A, Moriya K, Tanaka J, Tsukune Y, Komatsu N, Inokuchi K, Tamura H. Clinical impact of serum soluble SLAMF7 in multiple myeloma. Oncotarget. 2018;9(78):34784–93. 10.18632/oncotarget.26196. PMID: 30410677; PMCID: PMC6205184. 30410677 10.18632/oncotarget.26196 PMC6205184

[32] Assidi M. Strong prognostic value of SLAMF7 protein expression in patients with lymph node-positive breast cancer. Oncol Lett. 2022;24(6):433. 10.3892/ol.2022.13553. PMID: 36311690; PMCID: PMC9607863. 10.3892/ol.2022.13553 PMC9607863 36311690

[33] Deng Y, Zhang L, Dai C, Xu Y, Gan Q, Cheng J. SLAMF7 predicts prognosis and correlates with immune infiltration in serous ovarian carcinoma. J Gynecol Oncol. 2024;35(6):e79. 10.3802/jgo.2024.35.e79. Epub 2024 Mar 29. PMID: 38606823; PMCID: PMC11543254. 38606823 10.3802/jgo.2024.35.e79 PMC11543254

[34] Lingel H, Fischer L, Remstedt S, Kuropka B, Philipsen L, Han I, Sander JE, Freund C, Arra A, Brunner-Weinzierl MC. SLAMF7 (CD319) on activated CD8^+^ T cells transduces environmental cues to initiate cytotoxic effector cell responses. Cell Death Differ. 2025;32(3):561–72. 10.1038/s41418-024-01399-y. Epub 2024 Oct 10. PMID: 39390117; PMCID: PMC11893764. 39390117 10.1038/s41418-024-01399-y PMC11893764

[35] Fischer M. Conservation and divergence of the p53 gene regulatory network between mice and humans. Oncogene. 2019;38(21):4095–109. 10.1038/s41388-019-0706-9. Epub 2019 Feb 1. PMID: 30710145; PMCID: PMC6755996. 30710145 10.1038/s41388-019-0706-9 PMC6755996

[36] Han EH, Cho SH, Lee SN, Cho MY, Lee H, Lee SY, Ngoc Thi Tran C, Park HS, Min JY, Kim HM, Park MS, Kim TD, Lim YT, Hong KS. 3D Scaffold-Based Culture System Enhances Preclinical Evaluation of Natural Killer Cell Therapy in A549 Lung Cancer Cells. ACS Appl Bio Mater. 2024;7(11):7194–7206. 10.1021/acsabm.4c00800. Epub 2024 Oct 11. PMID: 39392900. 10.1021/acsabm.4c00800 39392900

[37] Zingoni A, Fionda C, Borrelli C, Cippitelli M, Santoni A, Soriani A. Natural Killer Cell Response to Chemotherapy-Stressed Cancer Cells: Role in Tumor Immunosurveillance. Front Immunol. 2017;8:1194. 10.3389/fimmu. 28993779 10.3389/fimmu.2017.01194 PMC5622151

[38] Ke M, Wang H, Zhou Y, Li J, Liu Y, Zhang M, Dou J, Xi T, Shen B, Zhou C. SEP enhanced the antitumor activity of 5-fluorouracil by upregulating NKG2D/MICA and reversed immune suppression via inhibiting ROS and caspase-3 in mice. Oncotarget. 2016;7(31):49509–26. 10.18632/oncotarget.10375. PMID: 27385218; PMCID: PMC5226525. 27385218 10.18632/oncotarget.10375 PMC5226525

[39] Cao G, Wang J, Zheng X, Wei H, Tian Z, Sun R. Tumor Therapeutics Work as Stress Inducers to Enhance Tumor Sensitivity to Natural Killer (NK) Cell Cytolysis by Upregulating NKp30 Ligand B7-H6. J Biol Chem. 2015;290(50):29964–73. 10.1074/jbc.M115.674010. Epub 2015 Oct 15. PMID: 26472927; PMCID: PMC4705966. 26472927 10.1074/jbc.M115.674010 PMC4705966

[40] Krześniak M, Zajkowicz A, Gdowicz-Kłosok A, Głowala-Kosińska M, Łasut-Szyszka B, Rusin M. Synergistic activation of p53 by actinomycin D and nutlin-3a is associated with the upregulation of crucial regulators and effectors of innate immunity. Cell Signal. 2020;69:109552. 10.1016/j.cellsig.2020.109552. Epub 2020 Feb 4. PMID: 32032660; PMCID: PMC7126238. 32032660 10.1016/j.cellsig.2020.109552 PMC7126238

[41] Baker SJ, Markowitz S, Fearon ER, Willson JK, Vogelstein B. Suppression of human colorectal carcinoma cell growth by wild-type p53. Science. 1990;249(4971):912-5. 10.1126/science.2144057. PMID: 2144057. 10.1126/science.2144057 2144057

