# Supplementary material for: The p53 tumor suppressor modulates the expression of proteins that control natural killer cell activity
Source: Cell Commun Signal. 2026 Mar 5;24:221. doi: 10.1186/s12964-026-02772-9 (PMC13072665; doi:10.1186/s12964-026-02772-9)
Supplement: Supplementary file 1 — Supplementary Material 1. [file 12964_2026_2772_MOESM1_ESM.docx]

| **Gene Symbol** | **Full Gene Name** | **Primer Sequences (5'→3')** | **Annealing Temp (°C)** | **Product Size (bp)** |
| --- | --- | --- | --- | --- |
| *ACTB* | Actin Beta | F: CAAATAAAGCCATGCCAATC | 56 | 144 |
|  |  | R: GCAAGCAGGAGTATGACGAG |  |  |
| *GAPDH* | Glyceraldehyde 3 Phosphate Dehydrogenase | F:TTCCATGGCACCGTCAAGGC | 63 | 173 |
|  |  | R:TGCAAATGAGCCCCAGCCTTCT |  |  |
| *NCR3LG1* | Natural Killer Cell Cytotoxicity Receptor 3 Ligand 1) | F: GTCTCCGTCAACTCTTTACGC | 67,5 | 188 |
|  |  | R: CTTTCAGATCACCTTCGGTCG |  |  |
| *SLAMF7* | SLAM Family Member 7 | F: GGAAGATCCAGCAAATACGG | 67,5 | 183 |
|  |  | R: GTTTTCTTTGGGCCGAGAAT |  |  |

Tabele. 1. Primer details for semi-quantitative PCR analysis.
